# Mapping the functional and structural connectivity of the scene network

**DOI:** 10.1002/hbm.26628

**Published:** 2024-02-20

**Authors:** David M. Watson, Timothy J. Andrews

**Affiliations:** ^1^ Department of Psychology and York Neuroimaging Centre University of York York UK

**Keywords:** diffusion MRI, fMRI, functional connectivity, naturalistic imaging, scene perception, structural connectivity

## Abstract

The recognition and perception of places has been linked to a network of scene‐selective regions in the human brain. While previous studies have focussed on functional connectivity between scene‐selective regions themselves, less is known about their connectivity with other cortical and subcortical regions in the brain. Here, we determine the functional and structural connectivity profile of the scene network. We used fMRI to examine functional connectivity between scene regions and across the whole brain during rest and movie‐watching. Connectivity within the scene network revealed a bias between posterior and anterior scene regions implicated in perceptual and mnemonic aspects of scene perception respectively. Differences between posterior and anterior scene regions were also evident in the connectivity with cortical and subcortical regions across the brain. For example, the Occipital Place Area (OPA) and posterior Parahippocampal Place Area (PPA) showed greater connectivity with visual and dorsal attention networks, while anterior PPA and Retrosplenial Complex showed preferential connectivity with default mode and frontoparietal control networks and the hippocampus. We further measured the structural connectivity of the scene network using diffusion tractography. This indicated both similarities and differences with the functional connectivity, highlighting biases between posterior and anterior regions, but also between ventral and dorsal scene regions. Finally, we quantified the structural connectivity between the scene network and major white matter tracts throughout the brain. These findings provide a map of the functional and structural connectivity of scene‐selective regions to each other and the rest of the brain.

## Graphical abstract

We determined the functional and structural connectivity profile of the scene processing network in the human brain. Our results indicate a bias between a posterior network (including the Occipital Place Area and posterior Parahippocampal Place Area [PPA]) implicated in processing egocentric visual features, and an anterior network (including the anterior PPA and Retrosplenial Complex) implicated in higher‐level mnemonic aspects of scene perception.

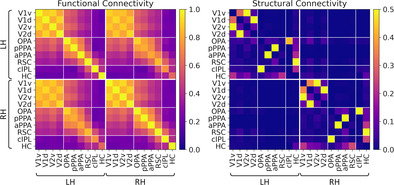

## 1 INTRODUCTION

Human observers are able to extract a wide variety of features from scenes, including contextual, spatial and navigational information. It is thought that the processing of such features critically depends on a core network of scene‐selective regions in the human brain (Epstein & Baker, 2019; Julian et al., 2018). This network comprises the Parahippocampal Place Area (PPA; Epstein & Kanwisher, 1998) on the ventral temporal surface, the Retrosplenial Complex (RSC; Maguire, 2001)—sometimes also referred to as the Medial Place Area (MPA; Silson et al., 2016)—on the medial parietal surface, and the Occipital Place Area (OPA; Dilks et al., 2013) on the lateral occipital surface. These core regions are complemented by a more extended network of regions implicated in higher‐level aspects of scene processing. Such regions include, but are not limited to, dorsal parietal areas implicated in visuospatial coding (Kravitz et al., 2011), medial temporal regions involved in memory and navigation (O'Keefe & Nadel, 1978) and medial parietal cortices implicated in representations of familiar environments (Silson et al., 2019).

The function of a given region depends not only on the tuning of neurons within the region itself, but also on how it connects with wider networks across the brain. Although the functional properties of scene‐selective regions have been researched extensively, less is known about how these regions connect with each other and the rest of the brain. Previous studies employing resting‐state functional connectivity have suggested a division between a posterior network including the OPA and posterior portions of the PPA, and an anterior network including the RSC and anterior portions of the PPA (Baldassano et al., 2013; Nasr et al., 2013; Silson et al., 2016; Tullo et al., 2023). The majority of studies have focussed on estimating functional connectivity between scene‐responsive regions of interest. In contrast, the connectivity of the core scene regions to much of the rest of the brain remains unclear. Our current understanding is that the posterior network connects more strongly with early visual regions and is proposed to primarily support processing of visual features within the immediately viewable scene. Meanwhile, the anterior network connects more strongly with extended scene regions such as the hippocampus, precuneus, posterior cingulate and caudal inferior parietal lobule and is proposed to support higher‐level aspects of scene processing such as navigation, recognition and memory recall (Baldassano et al., 2016). However, as almost all previous studies have measured functional connectivity at rest (although see Steel et al., 2021), it is less clear whether similar patterns are evident during naturalistic viewing (e.g., movie‐watching), which may capture more natural cognitive states in the brain (Finn, 2021). Thus, a comprehensive account of the functional connectivity of the core scene regions to each other and to the rest of the brain under different experimental paradigms remains lacking.

An alternative approach to measuring connectivity between regions is to measure the structural white matter connections. While functional connectivity measures the similarity of the time‐course of response between regions in a network, structural connectivity provides a more direct measurement of anatomical connectivity. Nevertheless, relatively little is known about the structural connectivity of the scene network. Studies of monkey anatomy have identified tracts connecting RSC to the medial temporal lobes, posterior cingulate, prefrontal cortices and caudal inferior parietal lobule (Kobayashi & Amaral, 2003, 2007; Kravitz et al., 2011). In humans, diffusion imaging studies have identified white matter tracts connecting the RSC to the hippocampus and prefrontal cortices (Greicius et al., 2009), and tracts connecting PPA and early visual cortices (Kim et al., 2006). We are not aware of any studies to date examining the structural connectivity of the OPA. Thus, a full account of the structural connectivity profile of the scene regions in humans is also lacking.

In this study, we aim to provide a comprehensive description of the functional and structural connectivity of the core scene regions to each other and the rest of the human brain. We leverage a combination of ‘in‐house’ and publicly available fMRI datasets to estimate functional connectivity across a range of movie‐watching and resting‐state paradigms. We then use a large diffusion imaging dataset from the Human Connectome Project to further measure structural connectivity between regions.

## 2 METHODS

### 2.1 Datasets

We used three MRI datasets—one dataset (Game of Thrones) was collected ‘in‐house’, and we obtained data from two further publicly available MRI datasets (StudyForrest and the Human Connectome Project).

#### 2.1.1 Game of Thrones

Our first dataset was collected ‘in‐house’—this dataset has been described previously (Noad et al., n.d., in review). We recruited 45 participants (15 male, 30 female, age range = 18–32, median age = 19) who were all neurologically healthy and had normal or corrected‐to‐normal vision. An additional 28 developmental prosopagnosics were also recruited, who are not analysed in the current study. Written consent was obtained from all participants and the study was approved by the ethics committee of the York Neuroimaging Centre (York, UK).

MRI data were collected at the York Neuroimaging Centre on a 3 T Siemens Magnetom Prisma scanner using a 64‐channel head coil. Functional data were acquired from 60 axial slices using a multiband EPI sequence (TR = 2 s, TE = 30 ms, FOV = 240 × 240 mm, matrix size = 80 × 80, 3 mm^3^ isotropic voxels, flip angle = 80°, anterior–posterior phase encoding direction, multiband acceleration factor = 2). Additional field‐map images were acquired in the same plane as the functional images (TR = 554 ms, short TE = 4.9 ms, long TE = 7.38 ms, flip angle = 60°, other parameters as per functional images). Finally, high‐resolution T1‐weighted anatomical images were acquired from 176 sagittal slices (TR = 2.3 s, TE = 2.26 ms, FOV = 256 × 256 mm, matrix size = 256 × 256, 1 mm^3^ isotropic voxels, flip angle = 8°).

Participants completed two functional scan runs. Participants first completed a movie‐viewing paradigm, in which they passively viewed short audiovisual clips (ranging from 50 to 117 s duration; total duration = 12 min 58 s) taken from the *Game of Thrones* television series. Videos were presented at the full resolution of the screen (1920 × 1080 pixels, subtending approximately 38.7° × 22.3° of visual angle). Secondly, participants completed a functional localiser scan. Images of faces, scenes and phase scrambled faces were presented in a block design. In each 9 s block, 4 images from a given condition were presented in sequence (600 ms duration, 200 ms ISI) followed by a 6 s blank period. A mid‐grey screen was displayed during the ISIs and blank periods. There were 9 blocks per condition (27 blocks total, 4 min 4 s scan duration). To maintain attention, participants performed an orthogonal task detecting occasional changes in the colour of the fixation cross, responding via a button press. Face images were sourced from the Radboud face database (Langner et al., 2010) and displayed on a grayscale 1/f amplitude mask background. Scene images were sourced from the SUN database (Xiao et al., 2010). All images subtended 8.4° of visual angle. In all scans, stimuli were back‐projected onto an in‐bore screen at a viewing distance of approximately 57 cm. Stimuli were presented in PsychoPy v3.1.5 (Peirce et al., 2019).

#### 2.1.2 StudyForrest

We next obtained movie‐watching, retinotopy and functional localiser MRI data for 15 participants from the publicly available StudyForrest dataset (Hanke et al., 2014, 2016; Sengupta et al., 2016). Briefly, in the movie‐watching paradigm, participants passively viewed and listened to approximately 2 h of the *Forrest Gump* movie. In retinotopy runs, the stimulus comprised a flickering chequerboard displayed within rotating wedge or expanding/contracting ring apertures. For the functional localiser, participants viewed images of human bodies, human faces, houses, inanimate objects, scenes and phase scrambled versions of those images. High‐resolution T1‐ and T2‐weighted anatomical images were also acquired. All data were acquired on a 3 T Philips Achieva MRI scanner.

#### 2.1.3 Human Connectome Project

Finally, we obtained resting‐state, movie‐watching, task and diffusion MRI data from the Human Connectome Project (HCP; Van Essen, Ugurbil, et al., 2012). We used a subset of 174 participants from the S1200 release who had fully completed all resting‐state and movie‐watching scans. Five participants had missing diffusion data and were omitted from structural connectivity analyses (leaving *n* = 169), and three participants had missing task data and were omitted from functional localiser analyses (leaving *n* = 171). A list of the participants is provided in Table S1.

Resting‐state and movie‐watching data were acquired on a 7 T Siemens Magnetom MRI scanner. The resting‐state data comprised four scan runs, each approximately 16 min in duration, in which participants viewed a fixation on a dark background with their eyes open. For the movie‐watching paradigm, participants passively viewed approximately 1 h of short audiovisual clips (split across four scan runs) taken from various Hollywood and independent movies (Cutting et al., 2012).

Task fMRI and diffusion data were acquired on a 3 T Siemens Skyra MRI scanner with a customised gradient coil. We obtained data from the working memory task to localise scene‐selective brain regions. Participants viewed images of human bodies, human faces, scenes and tools while performing a 0‐back or 2‐back recognition task. The diffusion data includes approximately 90 diffusion directions and 3 shells (*b* = 1000, 2000 and 3000 s/mm^2^). Full details of all HCP datasets can be found in the WU‐Minn HCP S1200 Data Release reference manual.

### 2.2 Pre‐processing

#### 2.2.1 Game of Thrones and StudyForrest

Data from the Game of Thrones and StudyForrest datasets followed the same pre‐processing pipeline. All pre‐processing was performed with FSL (Jenkinson et al., 2012).

Pre‐processing of functional data was performed with FEAT. For localiser data, pre‐processing included the following steps: motion correction using MCFLIRT (Jenkinson et al., 2002), slice‐timing correction, non‐brain removal using BET (Smith, 2002), spatial smoothing using a Gaussian kernel (FWHM = 6 mm, twice the voxel size), grand‐mean intensity normalisation by a single multiplicative factor and high‐pass temporal filtering. Pre‐processing of the StudyForrest retinotopy data followed the same steps but omitted the spatial smoothing. High‐pass temporal filter bandwidths were set at *σ* = 25, 24 and 50 s for the Game of Thrones localiser, StudyForrest localiser and StudyForrest retinotopy datasets respectively. For the StudyForrest datasets, the motion correction additionally registered each scan to a participant‐specific EPI template.

Movie‐watching data followed the same pre‐processing pipeline as the localiser plus two additional denoising steps. First, an ICA‐based denoising strategy was applied prior to the temporal filtering but following all other pre‐processing steps listed above. MELODIC (Beckmann & Smith, 2004) estimated spatiotemporal independent components from the data. The ICA‐AROMA toolbox (Pruim et al., 2015) was then used to automatically label noise components associated with head motion and regress them out of the data. This was done using an aggressive denoising strategy, such that all variance associated with the noise components was removed. High‐pass temporal filtering (*σ* = 50 s) was applied following the ICA denoising. A component‐based (CompCor) denoising strategy (Behzadi et al., 2007) was then applied to remove CSF‐related signals. FSL's FAST tool (Zhang et al., 2001) was used to derive tissue segmentations from the high‐resolution anatomical images (T1‐weighted for Game of Thrones, and T1‐ and T2‐weighted for StudyForrest). The CSF partial volume estimates were transformed to the functional volumes and thresholded at 90% to generate a mask. We obtained the mean and first four principal component timeseries from this CSF mask and regressed them out of the data.

Non‐brain removal of the high resolution T1 anatomical images was performed using BET. Functional images were co‐registered to the anatomical images via boundary‐based registration (Greve & Fischl, 2009). For the Games of Thrones dataset, the field‐maps were used to apply B0 unwarping to the functional images during the boundary‐based registration step. The anatomical images were then further registered to the MNI152 brain via a nonlinear registration by FNIRT (Andersson et al., 2010). Additionally, cortical surfaces and subcortical segmentations were generated from the anatomical images using Freesurfer (Dale et al., 1999).

#### 2.2.2 Human Connectome Project

All datasets were obtained following application of the HCP minimal pre‐processing pipeline (Glasser et al., 2013; Smith et al., 2013) including ICA‐based denoising. Briefly, this includes gradient distortion correction, motion correction, high‐pass temporal filtering (*σ* = 1000 s) and automated removal of noise components via FSL's FIX tool (Salimi‐Khorshidi et al., 2014). Subcortical data were nonlinearly registered to the MNI brain, and cortical data were registered to the fsLR32k standard surface (Van Essen, Glasser, et al., 2012) via a multimodal surface‐based alignment (MSMAll; Robinson et al., 2014, 2018) that incorporates information about cortical folding, resting‐state network and visuo‐topic maps, and areal features derived from myelin maps. The minimal pipeline incorporates some spatial smoothing—we applied additional smoothing (surface‐based for cortical data and volume‐based for subcortical data) to achieve an effective FWHM of 3.2 mm for resting‐state and movie‐watching data, and 4 mm for task data (twice the voxel resolution). For task data, an additional more stringent high pass temporal filter (*σ* = 100 s) was applied.

### 2.3 Regions of interest

#### 2.3.1 Early visual regions

We defined regions of interest (ROIs) for V1v, V1d, V2v and V2d. For the Game of Thrones and HCP datasets, ROIs were defined from the Benson retinotopic atlas (Benson et al., 2014). For the Game of Thrones dataset, ROIs were anatomically registered to each participant's cortical surface and restricted to the inner 19° of eccentricity (corresponding to half the visual angle of the movie stimulus). For the HCP datasets, we obtained pre‐calculated population receptive field (pRF) estimates for each participant (Benson et al., 2018). The Benson retinotopic atlas was then registered to each participant's surface via a Bayesian approach (implemented in the *neuropythy* toolbox) that incorporates information from the anatomy and pRF estimates to improve registration of the retinotopic maps (Benson & Winawer, 2018). The ROIs were then restricted to the inner 11° of eccentricity (corresponding to half the visual angle of the movie stimulus).

For the StudyForrest dataset, we defined ROIs manually from retinotopic maps in each participant. First, the unsmoothed retinotopy data were transformed onto the cortical surface. We then performed a travelling wave analysis using the *3dRetinoPhase* command in AFNI (Cox, 1996; Saad et al., 2001). The resulting polar angle phase maps were used to define ROIs by manually tracing along the phase reversals. For the Game of Thrones and StudyForrest datasets, all ROIs were transformed from the cortical surfaces to the functional volumes.

#### 2.3.2 Core scene regions

We functionally defined group‐level ROIs for the core scene‐selective regions—the OPA, RSC, and posterior and anterior divisions of the Parahippocampal Place Area (pPPA, aPPA). Data for the Game of Thrones and StudyForrest localiser scans were analysed with FEAT. Boxcar regressors were defined for each condition (Game of Thrones: faces, scenes and scrambled; StudyForrest: bodies, faces, houses, objects, scenes and scrambled) and convolved with a single‐gamma hemodynamic response function. These regressors, plus their temporal derivatives and six head motion confound regressors, were entered into a first‐level GLM analysis (Woolrich et al., 2001). Scene‐selective contrasts were defined as ‘scenes > (faces + scrambled)’ for the Game of Thrones dataset and ‘(scenes + houses) > faces’ for the StudyForrest dataset. For the StudyForrest dataset, parameter estimates were combined over scan runs by a higher‐level fixed effects analysis using FLAME (Woolrich et al., 2004). Individual estimates were then entered into a group‐level mixed‐effects analysis using FLAME. We used a clustering algorithm to define ROIs from the scene‐selective statistical maps. Seeds were defined at the peak voxels within each of the target regions, and the algorithm defined clusters of 250 spatially contiguous voxels (2000 mm^3^) around each of these seeds by iteratively adjusting the threshold till the target size was achieved (actual sizes varied slightly as an optimal solution was not always possible). In line with previous studies of the scene network (Baldassano et al., 2016), we divided the PPA region into posterior and anterior subdivisions. We split the full PPA region at the y‐axis coordinate that most evenly balanced the volumes of the subdivisions (~1000 mm^3^ each). The group level ROIs were then transformed from the MNI volume to each participant's functional volume.

We analysed the HCP task data using scripts from the HCP pipelines (https://github.com/Washington-University/HCPpipelines; Glasser et al., 2013), which internally use FEAT. In the working memory task, participants viewed images from different visual object categories while performing a 0‐back or 2‐back recognition task—by ignoring the task component, this data can be used to localise category selective regions. Eight boxcar regressors were defined for each condition (bodies, faces, scenes and tools in each task), and convolved with a double‐gamma hemodynamic response function. These were entered into a first‐level GLM analysis alongside their temporal derivatives. Twelve confound regressors, comprising head motion parameters and their temporal derivatives, were also included. Scene‐selective contrasts were defined as ‘scenes > faces’, collapsing across the tasks. Parameter estimates were combined over scan runs within each participant by a higher‐level fixed‐effects analysis, and then further over participants by a higher‐level mixed‐effects analysis using FLAME. We then used a surface‐based clustering algorithm to define clusters of spatially contiguous vertices (~500 mm^2^) around the peak vertices of each scene region. The PPA region was then split at the y‐axis coordinate that most evenly balanced the surface area of the posterior and anterior sub‐divisions (~250 mm^2^ each).

Figure S1 shows the scene‐selective contrasts and locations of scene ROIs in each dataset. A summary of the locations and size of each ROI is provided in Table S2, and a summary of the posterior and anterior PPA subdivisions is given in Table S3.

#### 2.3.3 Extended scene regions

We defined two additional extended scene regions suggested to show preferential connectivity with anterior core scene regions (Baldassano et al., 2016). In line with previous studies (Baldassano et al., 2013, 2016; Silson et al., 2016), we defined an ROI for the caudal Inferior Parietal Lobule (cIPL) from the PGp region of the JuBrain/SPM Anatomy Toolbox (Caspers et al., 2006; Eickhoff et al., 2005). The cIPL and OPA regions occasionally overlapped slightly—in these cases the overlap was removed from both masks. The cIPL masks were then transformed from the MNI volume to each participant's functional volume (Game of Thrones and StudyForrest) or fsLR32k surface (HCP). We also defined participant‐specific ROIs for the hippocampus using Freesurfer's automatic subcortical segmentation of the anatomical images (Fischl et al., 2002). These were then transformed to each participant's functional volume (Game of Thrones and StudyForrest) or MNI volume (HCP).

### 2.4 Functional connectivity

For each dataset the pre‐processed and denoised functional timeseries were converted to units of percent signal change. In the StudyForrest and HCP datasets, where there are multiple scan runs, the timeseries were concatenated over runs. Timeseries were then averaged over grayordinates within each ROI.

We first measured functional connectivity between the early visual (V1 − V2v/d), core scene (OPA, pPPA, aPPA, RSC) and extended scene regions (cIPL, hippocampus). Timeseries were correlated between all pairings of ROIs across both hemispheres. Correlations were converted to units of Fisher's z then averaged over participants. To provide additional visualisations, we applied multidimensional scaling and hierarchical clustering. The group average correlation matrix was converted back to units of Pearson's r then converted to correlation distances. This was then submitted to metric multidimensional scaling (implemented in *scikit‐learn*; Pedregosa et al., 2011) and hierarchical clustering using an unweighted average distance method (implemented in *scipy*; Virtanen et al., 2020).

We further examined connectivity between the core scene regions (OPA, pPPA, aPPA, RSC) and the rest of the brain. We first employed a seed‐based approach—average timeseries for each core scene ROI were correlated with all grayordinates throughout the rest of the brain. The resulting whole‐brain correlation maps were converted to Fisher's z, and then submitted to group analyses using a permutation testing procedure implemented with the PALM toolbox (Winkler et al., 2014). Sign‐flip permutations were used to perform: (1) one‐sample tests of correlations for each seed region against zero and (2) paired‐samples tests of correlations between seed regions. We generated Threshold Free Cluster Enhanced (TFCE; Smith & Nichols, 2009) images, then used a maximum permutation statistic approach to derive *p*‐values controlling for the FWER over grayordinates and positive/negative contrasts (Alberton et al., 2020). For the HCP data, the permutation tests additionally controlled the FWER over left and right surfaces (Winkler, Webster, et al., 2016), and a further Bonferroni correction was applied for two comparisons over the surface and subcortical structures. We ran 1000 permutations for each comparison, and *p*‐values were derived from a gamma function approximation of the permutation distribution (Winkler, Ridgway, et al., 2016).

To provide a summary of the connectivity with the rest of the brain, we additionally measured connectivity with 17 resting‐state networks across the cortex (Yeo et al., 2011). To prevent double‐dipping, any overlap between a given scene and network region was removed from the network region prior to further analysis. Timeseries were averaged over grayordinates within each of the 17 networks in each hemisphere then correlated with the average timeseries for the core scene regions. We also employed the same approach to examine connectivity with nine subcortical structures (amygdala, caudate nucleus, cerebellar cortex, hippocampus, nucleus accumbens, pallidum, putamen, thalamus and ventral diencephalon). These structures were defined from each participant's anatomical image using Freesurer's automatic subcortical segmentation (Fischl et al., 2002). Note that the hippocampus region is identical to the one included in the extended scene ROIs. Timeseries were averaged over voxels within each subcortical structure and correlated with the core scene regions.

### 2.5 Structural connectivity

All diffusion data were obtained after processing by the HCP diffusion analysis pipeline (Glasser et al., 2013) including distortion correction with EDDY and modelling of crossing fibres with BEDPOSTX. We then conducted probabilistic tractography analyses using PROBTRACKX2. All surface ROIs were transformed from the fsLR32k surface to each participant's diffusion volume and restricted to the grey matter ribbon. In all analyses we included the pial surface as a termination mask, such that streamlines would be terminated and retained upon contact—this helps prevent anatomically implausible streamlines, for instance crossing between sulci or the medial surfaces of the hemispheres. A volume mask of all subcortical grey matter was also included as a ‘wtstop’ termination mask, such that streamlines could enter the mask but would be terminated and retained upon attempting to leave. Finally, a mask of the ventricles was included as an exclusion mask—any streamlines contacting this would be terminated and excluded. All tractography analyses employed modified Euler streamlining, loop‐checking, and the following parameters: maximum steps = 2000, step length = 0.5 mm, curvature threshold = ±80°, minimum streamline length = 5 mm and subsidiary fibre volume fraction threshold = 0.01.

We first measured structural connectivity between the early visual (V1 − V2v/d), core scene (OPA, pPPA, aPPA, RSC), and extended scene regions (cIPL, hippocampus). This entailed running PROBTRACKX2 in its ‘network’ mode. Each region in turn was chosen as a seed, and 5000 streamlines were seeded from each voxel within it. All other regions were included as waypoint masks, so that only streamlines which hit at least one of the other regions were retained. Repeating this for each seed region generates a square connectivity matrix counting the number of streamlines from each seed region hitting each of the other target regions. Each row of this matrix was then normalised by the ‘waytotal’ (the total number of valid streamlines) for the corresponding seed region, yielding a matrix of connection probabilities representing the proportion of valid streamlines from each seed region hitting each of the other target regions. These probability matrices were then averaged over participants. We additionally submitted the group average matrix to multidimensional scaling and hierarchical clustering by subtracting the probabilities from one to convert to a distance metric and averaging over the diagonal to render the matrix symmetrical.

We next performed seed‐based tractography analyses to estimate structural connectivity over the whole brain. A given core scene region was selected as the seed, and 5000 streamlines were seeded from each voxel within it. No waypoint masks were included. This produced a whole brain map of streamline counts which was then normalised by the waytotal to generate a map of connection probabilities. Each of these probability maps were then transformed to the MNI space and entered into group permutation analyses using the PALM toolbox. This proceeded as described for the functional seed connectivity analyses. Sign‐flip permutations were used to conduct one‐sample tests of the connection probabilities against zero and paired‐sample tests of the connection probabilities between regions. TFCE statistic images were generated, and corresponding FWER‐corrected *p*‐values derived using a maximum permutation statistic approach and a gamma approximation of the permutation distribution.

To provide a summary of grey‐to‐grey matter structural connectivity over the whole brain, we next performed tractography analyses between the core scene regions and the 17 resting‐state networks (Yeo et al., 2011) and subcortical structures (Fischl et al., 2002). The resting‐state networks were projected from the fsLR32k surface to each participant's diffusion volume and restricted to the cortical ribbon. A given scene region was entered as the seed region, seeding 5000 streamlines from each voxel, and all resting‐state networks in both hemispheres were entered as both target and waypoint masks. This ensured that only streamlines hitting at least one of the network regions were retained. Any overlap between the seed and network regions was removed from the network regions beforehand. We measured the number of streamlines hitting each target region and normalised by the waytotal to produce a vector of connection probabilities from the seed to each network region. The same procedure was used to measure connection probabilities from each scene region to the subcortical structures. The resulting seed‐to‐target connection probability matrices were averaged over participants.

Finally, to provide a summary of grey‐to‐white matter structural connectivity, we measured connectivity between the core scene regions and major white matter tracts using the XTRACT toolbox (Warrington et al., 2020). In an initial stage, probabilistic tractography analyses were applied to each participant's diffusion data to generate individualised reconstructions of 42 white matter tracts (19 lateralised tracts in each hemisphere plus 4 interhemispheric tracts). In a second stage, connectivity blueprints are generated estimating tract termination maps across the cortical surface. A whole‐brain tractography analysis was run using the white/grey‐matter boundary surface as a seed mask, seeding 1000 streamlines per vertex, and the whole brain white‐matter as a target mask. This generated a matrix of streamline counts from each surface vertex to each white matter voxel. A corresponding connectivity matrix was generated between the tracts identified in the first stage and all white‐matter voxels. Taking the product of these two matrices yields a vertex‐by‐tracts matrix, representing whole‐brain connectivity blueprints for each tract. High loadings between a given vertex and tract indicate a high probability of that tract terminating at that location on the white/grey‐matter boundary. For each tract, we averaged the blueprint loadings over vertices within each of the core scene ROIs, then further averaged over participants. This produced an ROIs‐by‐tracts matrix indicating the structural connectivity between each scene region and tract.

## 3 RESULTS

### 3.1 Functional connectivity

**FIGURE 1:**
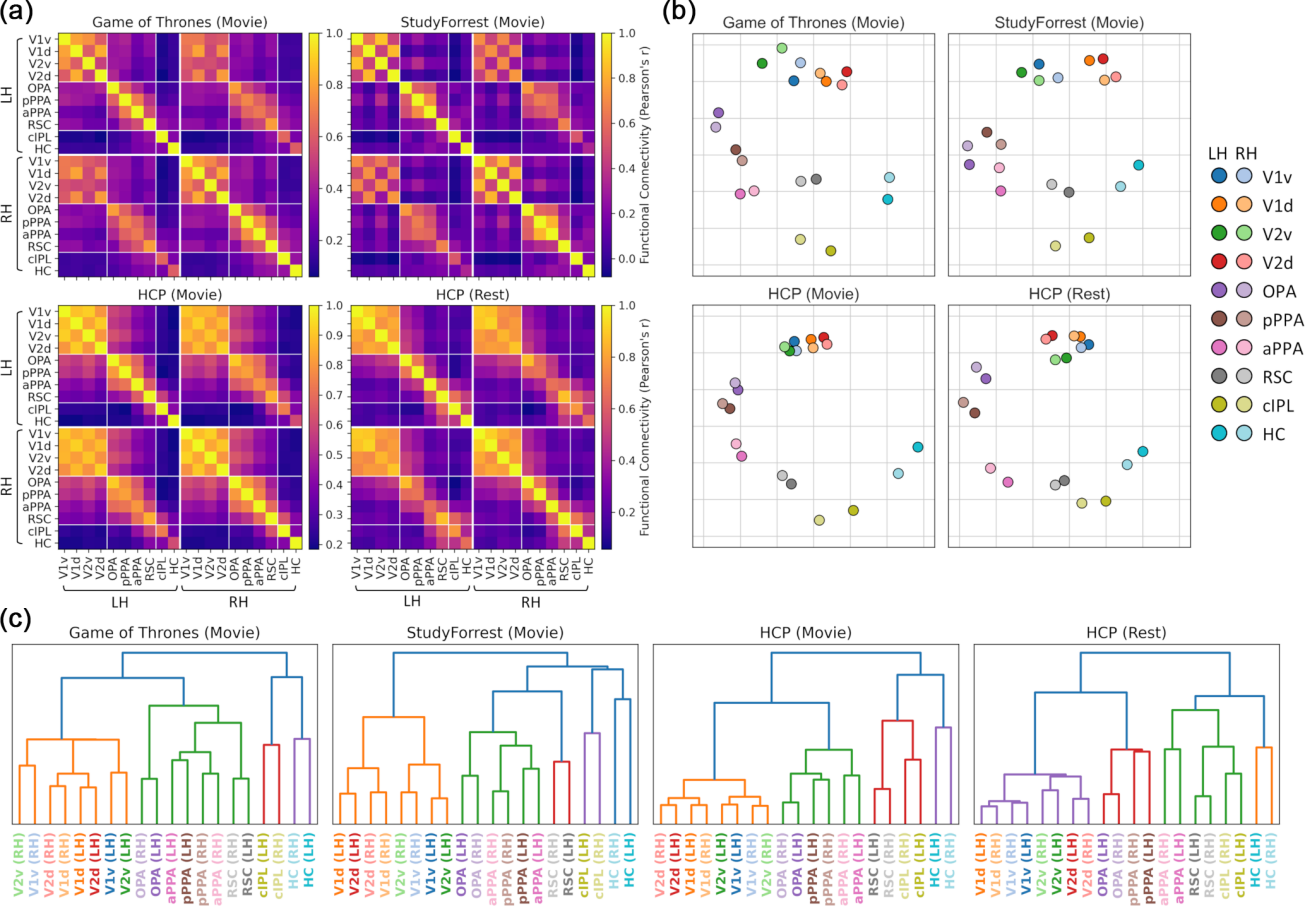
Functional connectivity between early visual and core and extended scene regions for Game of Thrones, StudyForrest, and HCP movie‐watching and resting‐state datasets. (a) Group average correlation matrices for each dataset. (b) Multidimensional scaling and (c) hierarchical clustering representations of group average correlation matrices.

We first examined the functional connectivity profile of the scene network using three movie‐watching (Game of Thrones, StudyForrest and HCP) datasets plus a resting‐state dataset from the HCP. To begin, we measured connectivity between early visual (V1 − V2v/d), core scene (OPA, pPPA, aPPA, RSC), and extended scene (cIPL, hippocampus) regions of interest included in Baldassano et al.'s (2016) model. Timeseries were averaged over grayordinates within each region and then correlated between all pairwise combinations of regions. Group average connectivity matrices are illustrated in Figure 1a. Connectivity often appeared stronger within than between each group of regions (early visual, core scene and extended scene). Within the scene network, the results supported a bias between a posterior scene network including the OPA which connected more strongly with early visual regions, and an anterior scene network including the RSC and connecting more with the extended regions, with each network converging in the PPA. The overall pattern of connectivity appeared similar both within and between hemispheres, indicating clear interhemispheric connectivity. The pattern of connectivity also appeared similar across datasets.

To provide an alternative visualisation, we applied multidimensional scaling to the group average connectivity matrices (Figure 1b). This generates a two‐dimensional space where regions which are more strongly connected appear closer together, and regions which are less strongly connected appear further apart. Homotopic regions between hemispheres appeared close together, reflecting strong interhemispheric connectivity. Early visual regions emerged as a cluster near the top of the space, extended scene regions appeared in the bottom‐right, with core scene regions in between. OPA and pPPA typically appeared closer to the early visual regions, while aPPA and RSC emerged closer to the extended scene regions, supporting a bias between posterior and anterior scene networks which converge in the PPA. All core scene regions were also well connected with one another, although a clearer division between posterior and anterior PPA emerged in the HCP resting‐state dataset than the other movie‐watching datasets.

A further visualisation was derived using hierarchical clustering (Figure 1c). The most immediate pairings occurred between homotopic regions in each hemisphere, again highlighting the interhemispheric connectivity. The early visual, core scene and extended scene groups each organised into three broad branches, although the representation of the core scene regions was somewhat variable over datasets. In the Game of Thrones and StudyForrest datasets, the anterior and posterior PPA paired together most immediately, with the OPA and RSC joining the core scene branch later. In the HCP movie‐watching dataset, the posterior PPA and OPA paired together first then later joined with the anterior PPA, while the RSC emerged within the extended scene region branch. The distinction between posterior and anterior networks appeared clearer in the HCP resting‐state dataset—the posterior PPA and OPA again paired together, while now both the anterior PPA and RSC joined the extended scene region branch.

In general, these results support a bias between a posterior scene network that is more connected with early visual regions, and an anterior network that is more connected with extended scene regions, with each network converging in the PPA. Nevertheless, the scene regions also all appeared well connected to each other, suggesting against a hard division between posterior and anterior networks. The distinction between networks appeared greater in the HCP resting‐state dataset than in the other movie‐watching datasets. Previous studies have primarily employed resting‐state functional connectivity and hence may have estimated a larger distinction between the networks than would have been obtained during naturalistic viewing.

**FIGURE 2:**
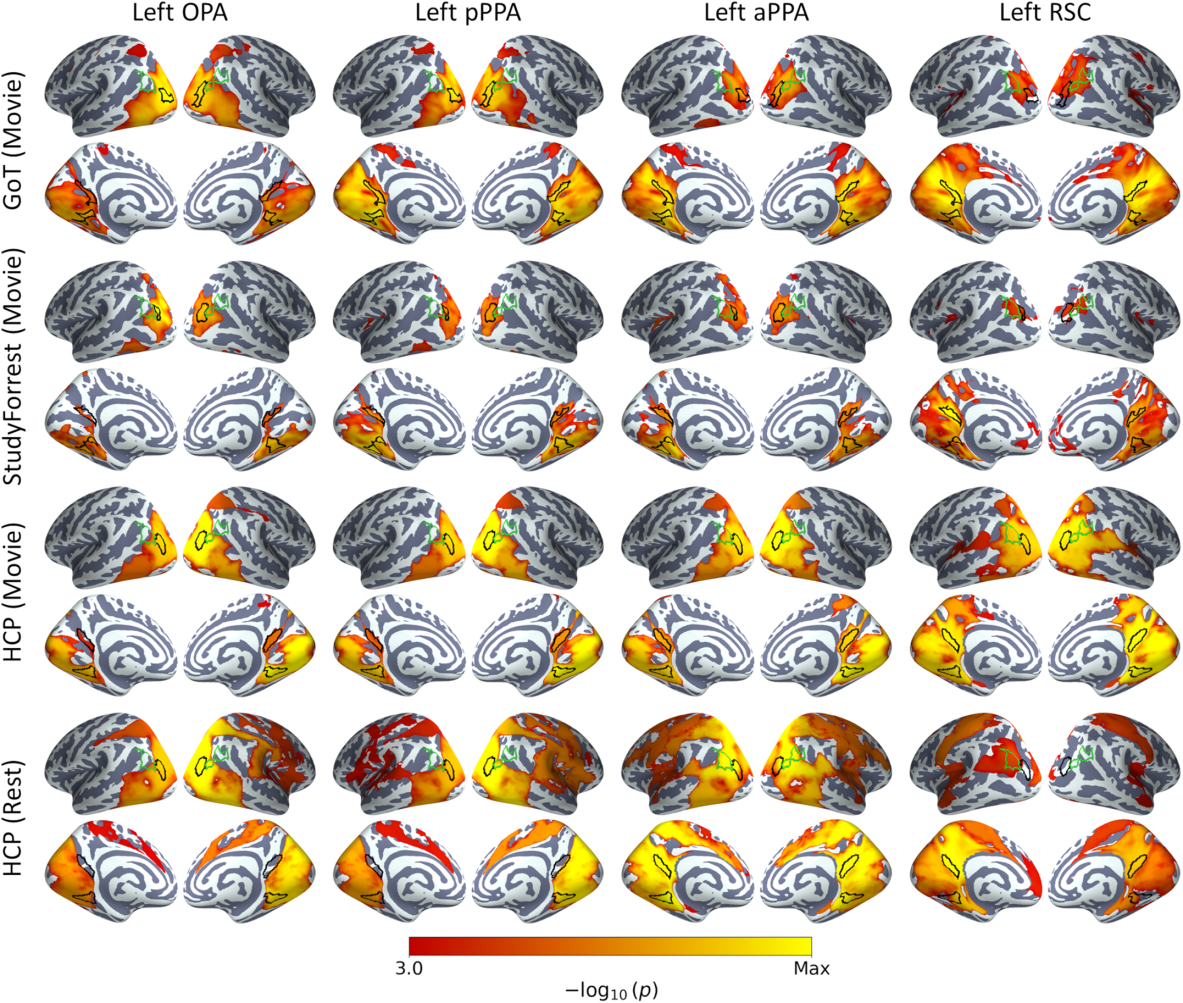
Seed‐based functional connectivity between left hemisphere core scene regions and cortical grey matter. Statistical overlays illustrate FWER‐corrected TFCE *p*‐values for one‐sample tests of functional connectivity correlations against zero over participants. Annotations indicate locations of core scene (OPA, PPA, RSC; black outlines) and cIPL (green outlines) regions.

Our previous analyses were restricted to a preselected set of early visual and scene ROIs. We next adopted a number of approaches to examine connectivity between the core scene regions and the rest of the brain. We first conducted seed‐based functional connectivity analyses between the core scene regions and the rest of the cerebral cortex. Average timeseries for each core scene region were correlated with all cortical grayordinates. Cortical connectivity maps for the left hemisphere seeds are displayed in Figure 2, and for right hemisphere seeds in Figure S2. This revealed extensive interhemispheric connectivity, and indeed the pattern of connectivity appeared highly similar in each hemisphere and between homotopic seeds. Within the HCP dataset, more extensive connectivity was observed during resting‐state than movie‐watching, consistent with previous literature (Betti et al., 2013; Lynch et al., 2018; Wang et al., 2017). Each of the core scene regions displayed clear connectivity with each other, but also with many regions outside of the scene network.

**FIGURE 3:**
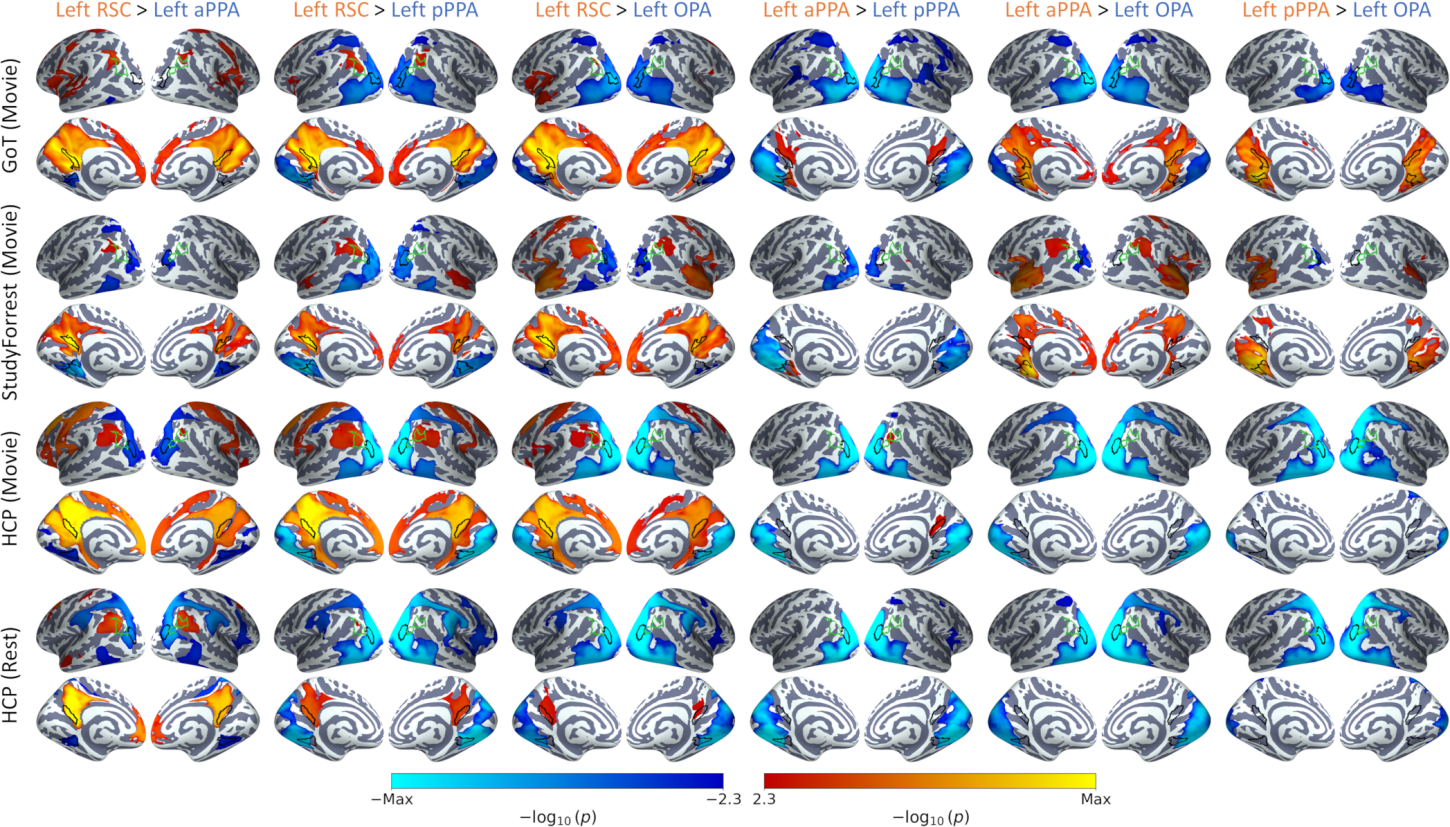
Contrasts of seed‐based functional connectivity measured between left hemisphere core scene regions and cortical grey matter. Statistical overlays illustrate FWER‐corrected TFCE *p*‐values for paired‐sample tests of functional connectivity correlations between seed regions over subjects. Annotations indicate locations of core scene (OPA, PPA, RSC; black outlines) and cIPL (green outlines) regions.

To compare the patterns of connectivity between different scene regions, we contrasted the connectivity maps. Contrasts between left hemisphere seed regions are shown in Figure 3, and for right hemisphere seeds in Figure S3. Unthresholded contrasts are displayed in Figures S4 and S5. For each pair of seeds, the more anterior region is represented in the positive end of the contrast, such that positive and negative values would be expected to highlight biases towards more anterior and posterior regions respectively. The contrasts indicated greater connectivity between the anterior network and many brain regions implicated in higher‐level mnemonic processes, including lateral inferior parietal areas (encompassing the cIPL), medial parietal, medial temporal, medial frontal and dorsolateral frontal cortices. Meanwhile, the posterior network appeared more strongly connected with regions implicated in visual perception, including early visual, ventral temporal and posterior parietal regions. Finally, the pattern of connectivity appeared broadly consistent between datasets—this is most clearly seen in the unthresholded contrasts (Figures S4 and S5).

**FIGURE 4:**
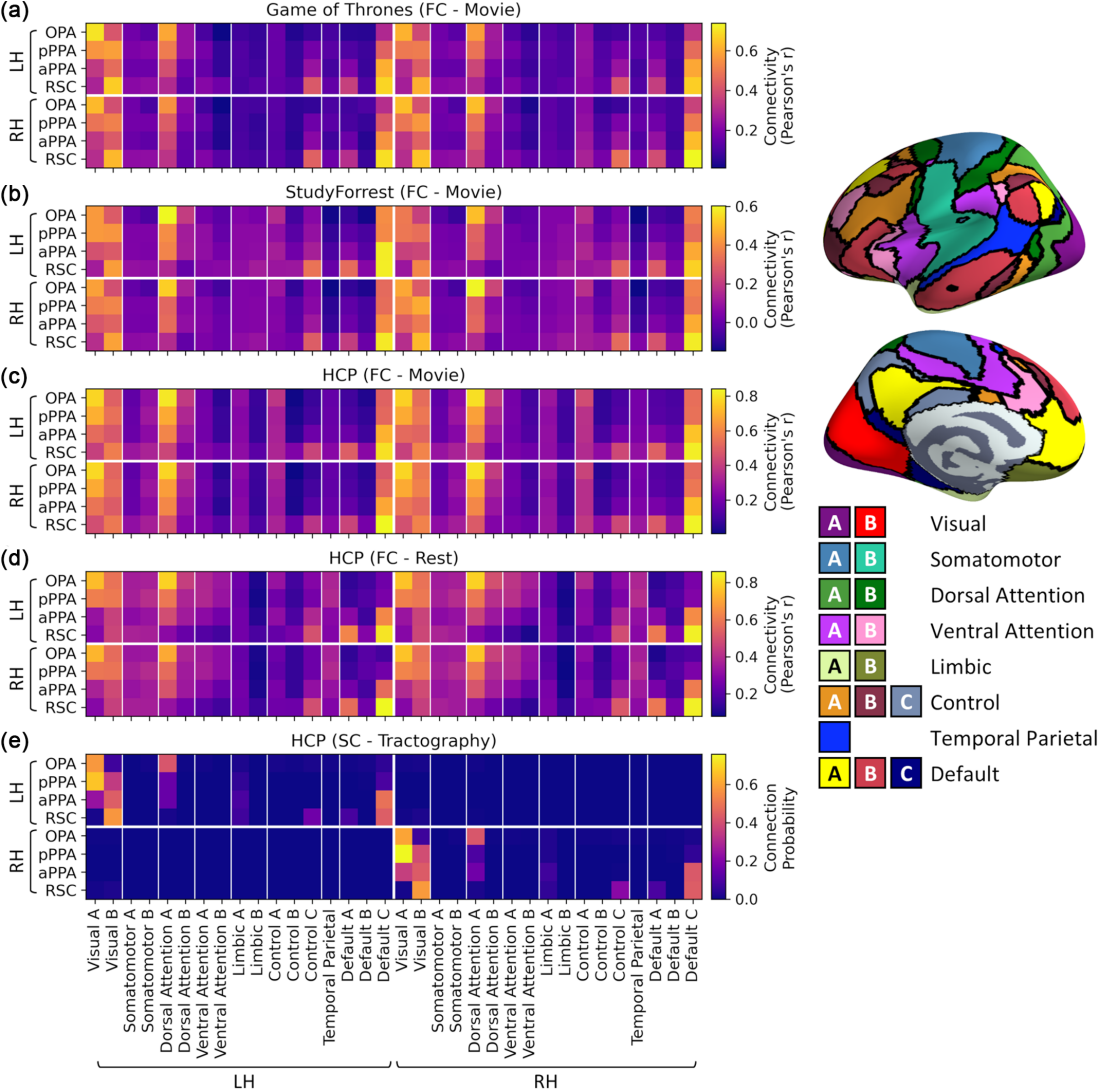
Connectivity between core scene regions and 17 resting‐state networks (Yeo et al., 2011). Group average matrices illustrate functional connectivity for (a) Game of Thrones, (b) StudyForrest, (c) HCP movie‐watching, (d) HCP resting‐state datasets and (e) structural connectivity for the HCP dataset. Locations of networks are illustrated on the right.

To provide a summary of the cortical connectivity over different brain regions, we also measured connectivity between the core scene regions and 17 cortical resting‐state networks (Yeo et al., 2011). The average timeseries for each core scene region was correlated with the timeseries averaged over all grayordinates within each of the 17 networks in both hemispheres (after removing any overlap with the scene region from the network region). Figure 4a–d shows the correlation matrix for each dataset, and contrasts between regions are displayed in Figure S6a–d. As before, highly similar patterns of connectivity were observed within and between hemispheres, demonstrating clear interhemispheric connectivity. The connectivity patterns also appeared consistent between the datasets. Networks showing stronger connectivity with posterior (OPA, pPPA) than anterior scene regions (aPPA, RSC) included Visual A (which includes posterior ventral and lateral occipital visual cortices) and Dorsal Attention A (which includes lateral occipital and posterior parietal cortices). Meanwhile, the Control C, Default A and Default C networks (which include medial temporal, lateral and medial parietal and ventromedial and dorsolateral prefrontal cortices) consistently showed stronger connectivity with anterior than posterior scene regions.

**FIGURE 5:**
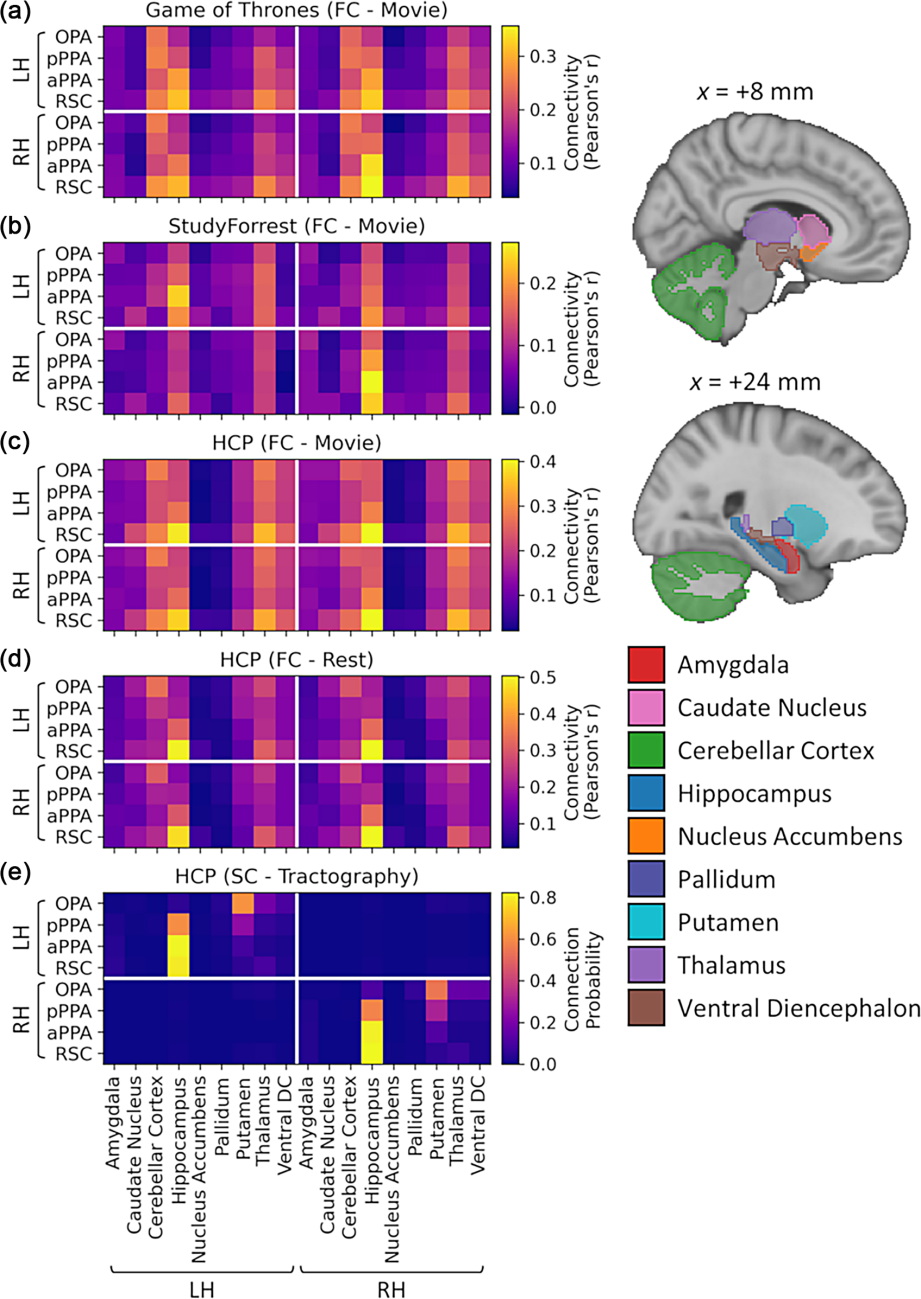
Connectivity between core scene regions and subcortical structures. Group average matrices illustrate functional connectivity for (a) Game of Thrones, (b) StudyForrest, (c) HCP movie‐watching, (d) HCP resting‐state datasets and (e) structural connectivity for the HCP dataset. Locations of structures are illustrated on the right.

Finally, we examined functional connectivity with subcortical structures. The results of seed‐based connectivity analyses within the subcortical structures are displayed in Figures S7 and S8, and thresholded and unthresholded contrasts between regions are shown in Figures S9–12. These highlighted bilateral clusters of connectivity most consistently in the hippocampus, thalamus and cerebellum. Anterior scene regions showed preferential connectivity with the hippocampus and, less consistently, the thalamus. Multiple clusters were observed in the cerebellum, variously displaying preferential connectivity with both posterior and anterior scene regions. To provide a summary of the subcortical connectivity, we measured connectivity between the core scene regions and each of the subcortical regions in Freesurfer's Aseg atlas (Fischl et al., 2002). The correlation matrices for each dataset are shown in Figure 5a–d, and contrasts between regions are shown in Figure S13a–d. As before, extensive interhemispheric connectivity was observed, and the pattern of connectivity appeared similar within and between hemispheres. Connectivity patterns also appeared consistent between datasets. Results for the hippocampus are duplicated from the main ROI analyses (Figure 1a)—this showed preferential connectivity with more anterior regions (aPPA and RSC). The core scene regions also displayed relatively high connectivity with the thalamus and cerebellum, although this was less consistent over datasets and did not show a reliable bias towards the posterior or anterior networks.

Taken together, these results demonstrate that the bias between the posterior and anterior scene networks extends to their connectivity with many other cortical and subcortical regions throughout the brain. The anterior network showed preferential connectivity with the default mode and cognitive control networks and the hippocampus. Meanwhile the posterior network showed preferential connectivity with early visual regions, but also with posterior parietal regions indicating a possible role for this network in the dorsal visual stream.

### 3.2 Structural connectivity

**FIGURE 6:**
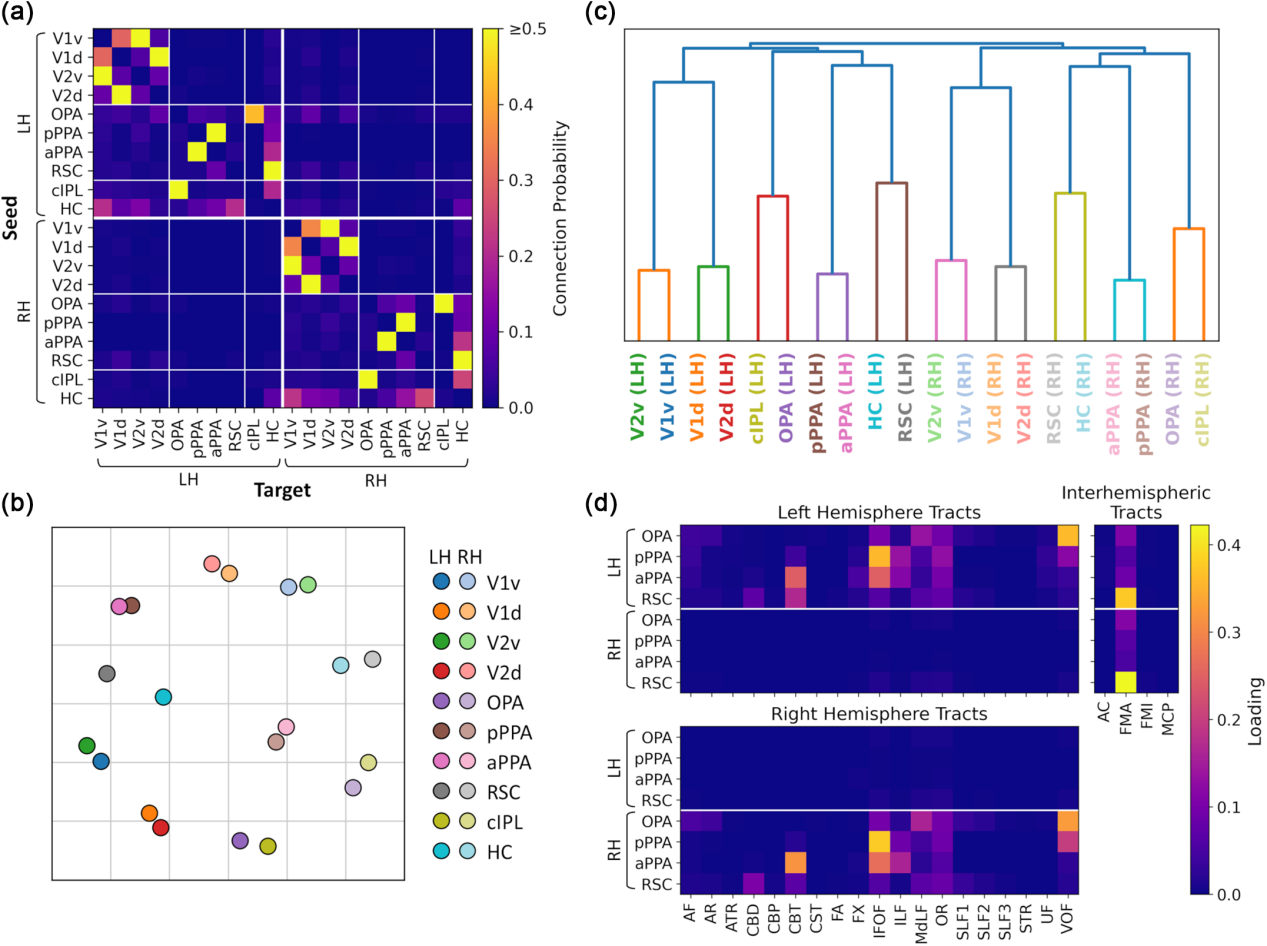
Tractography results between early visual and core and extended scene regions. (a) Group average connection probabilities from each seed region to all other target regions. The upper limit of the colourmap has been saturated to better illustrate the structure of the matrix. (b) Multidimensional scaling and (c) hierarchical clustering representations of group average connectivity matrix. (d) Connectivity blueprints between core scene regions and 42 white matter tracts identified with the XTRACT toolbox. Higher loadings indicate a greater probability of that tract terminating within the region. AC, anterior commissure; AF, arcuate fasciculus; AR, acoustic radiation; ATR, anterior thalamic radiation; CBD, CBP, CBT, cingulum bundle—dorsal, perigenual and temporal subsections; CST, corticospinal tract; FA, frontal aslant; FMA, forceps major; FMI, forceps minor; FX, fornix; IFOF, inferior fronto‐occipital fasciculus; ILF, inferior longitudinal fasciculus; MCP, middle cerebellar peduncle; MdLF, middle longitudinal fasciculus; OR, optic radiation; SLF1‐3, superior longitudinal fasciculus 1–3; STR, superior thalamic radiation; UF, uncinate fasciculus; VOF, vertical occipital fasciculus.

We next employed probabilistic tractography analyses of diffusion data from the HCP to examine the structural connectivity of the scene network. The results supported some distinctions between anterior and posterior scene regions, while also indicating some ventral versus dorsal differences. We first estimated connectivity between the early visual and core and extended scene regions. Streamlines were seeded from each region in turn, and connection probabilities are given by the proportion of valid streamlines hitting each of the other target regions. Figure 6a shows the group average connectivity matrix. While the pattern of connectivity appeared similar in each hemisphere, the majority of connections appeared to fall within rather than between hemispheres—in contrast to the functional connectivity. In addition, the pattern of structural connectivity appeared more heavily influenced by the anatomy than was the case for the functional connectivity. In some cases, this still supported the distinction between the posterior and anterior scene regions—for instance a greater proportion of connections were observed between the hippocampus and anterior (aPPA, RSC) than posterior (OPA, pPPA) scene regions. Other cases did not follow this trend however—for instance there was a high connection probability between OPA and cIPL due to their anatomical proximity, despite these regions showing minimal functional connectivity. To provide an alternative visualisation, we submitted the connectivity matrix to multidimensional scaling (Figure 6b) and hierarchical clustering (Figure 6c). Each of these indicated a clear divide between the hemispheres, with the left and right hemispheres emerging on opposite sides of the multidimensional scaling space and in separate branches of the hierarchical clustering. A broad distinction was also observed between early visual regions versus core and extended scene regions. However, there was less distinction between the core and extended scene regions themselves—the OPA most immediately paired with the cIPL, the RSC paired with hippocampus, and the anterior and posterior PPA paired together. Thus, the structural connectivity between the preselected regions of interest showed less evidence of a distinction between the posterior and anterior scene networks than was seen in the functional connectivity.

**FIGURE 7:**
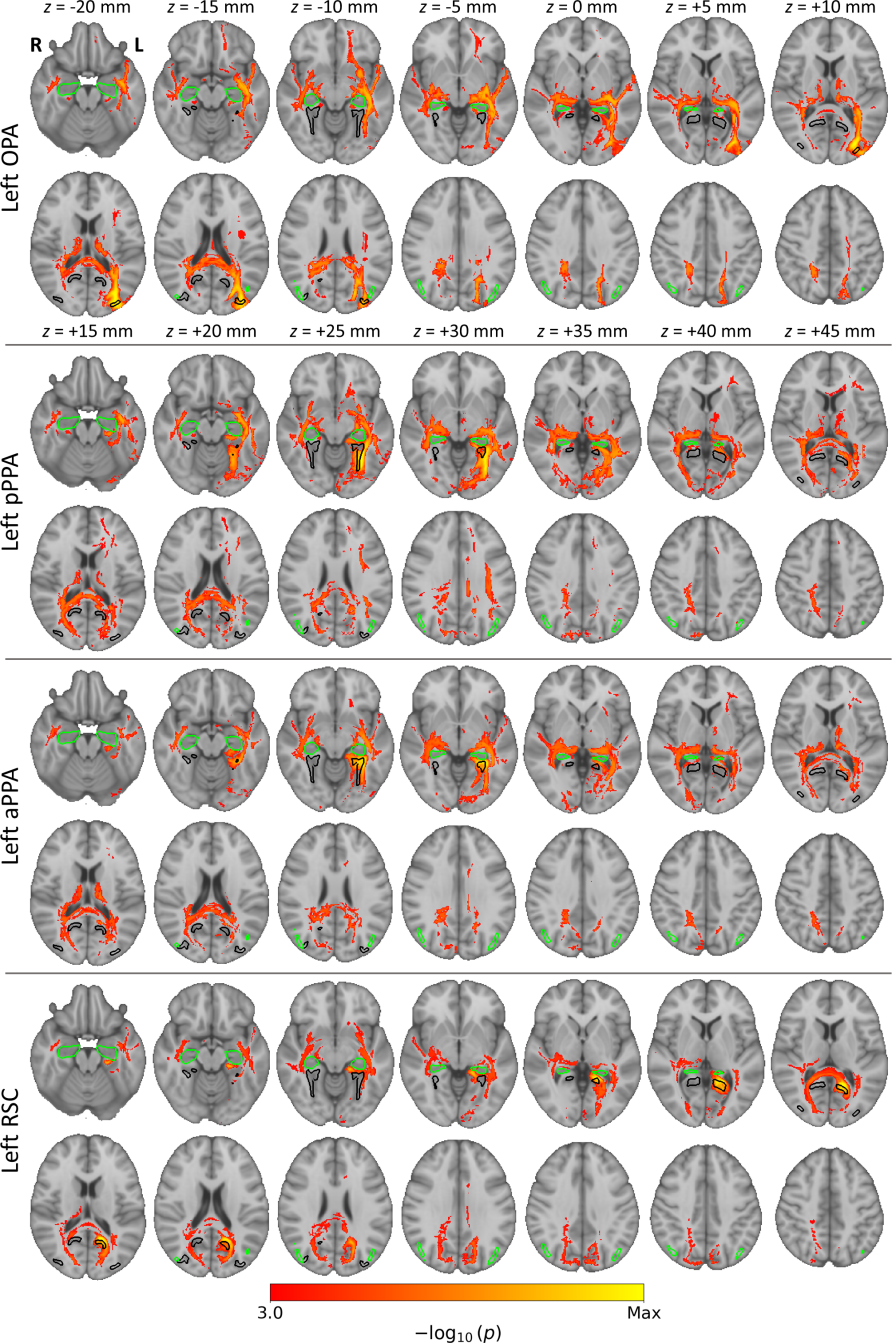
Seed‐based tractography from left hemisphere core scene regions. Statistical overlays illustrate FWER‐corrected TFCE *p*‐values for one‐sample tests of connection probabilities against zero over participants. Annotations indicate locations of core (OPA, PPA, RSC; black outlines) and extended (cIPL, hippocampus; green outlines) scene regions.

**FIGURE 8:**
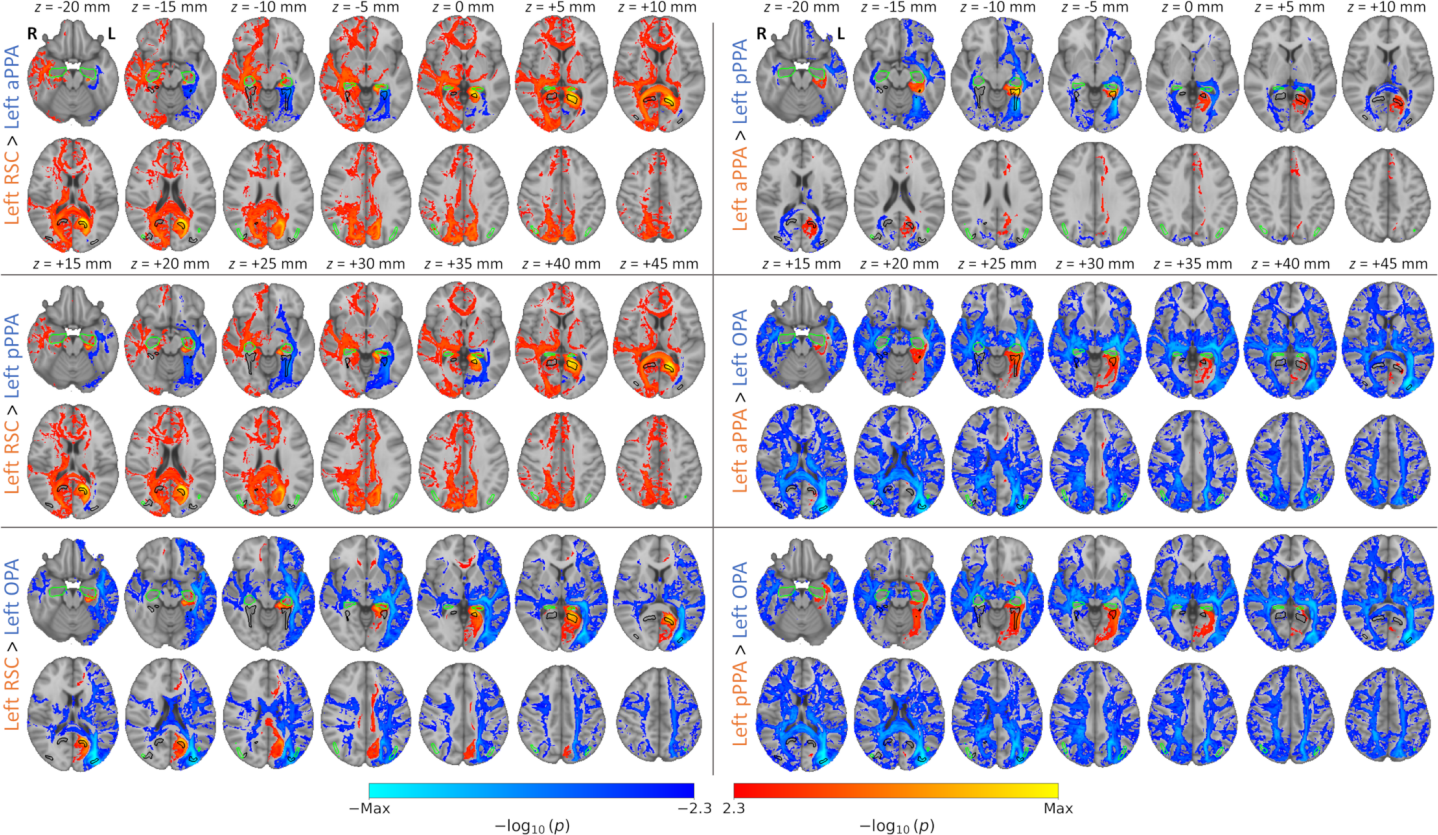
Contrasts of seed‐based tractography between left hemisphere core scene regions. Statistical overlays illustrate FWER‐corrected TFCE *p*‐values for paired‐sample tests of connection probabilities between seed regions over subjects. Annotations indicate locations of core (OPA, PPA, RSC; black outlines) and extended (cIPL, hippocampus; green outlines) scene regions.

We next examined the structural connectivity between scene regions and the rest of the brain. We first performed whole‐brain tractography analyses—streamlines were seeded from each of the core scene regions, and connection probabilities were estimated for all voxels throughout the brain. These connection probability maps were then combined over subjects by contrasting the probabilities against zero or between seed regions. Group‐level statistical maps for the left hemisphere seeds are shown in Figure 7, and for right hemisphere seeds in Figure S14. Three dimensional renderings of these maps are shown in Video S1. All of the scene regions showed high densities of tracts extending anteriorly along the temporal lobe, posteriorly into the occipital lobes, and superiorly through parietal cortices. Many interhemispheric tracts were also observed passing through the splenium. Contrasts between left hemisphere seeds are shown in Figure 8, and for right hemisphere seeds in Figure S15. Animations of these contrasts are shown in Videos S2 and S3, and unthresholded contrasts are shown in Figures S16 and S17. Unlike the functional connectivity, the contrasts more clearly emphasised a distinction between dorsal and ventral regions, rather than posterior and anterior. The more ventral anterior and posterior PPA generally showed a greater density of tracts passing along the temporal lobe. Meanwhile, the more dorsal RSC and OPA showed greater densities of tracts in parietal and frontal regions, as well as interhemispheric connections.

We next sought to provide summaries of the whole‐brain structural connectivity profiles. We first quantified grey‐matter to grey matter connections by performing tractography analyses seeding from the core scene regions to the 17 cortical resting‐state networks (Yeo et al., 2011) or to the subcortical structures (Fischl et al., 2002). Streamlines were seeded from each core scene region in turn, and connection probabilities were given by the proportion of valid streamlines hitting each of the target network/subcortical regions. In the case of the resting‐state networks, any overlap with the seed region was first removed from the network regions. Figure 4e shows the results of tractography analyses seeding from each of the scene regions to the resting‐state networks, and contrasts between regions are displayed in Figure S6e. Although there were relatively few interhemispheric connections, the structural connectivity within each hemisphere did show some resemblance to the functional connectivity patterns. Posterior scene regions (OPA, pPPA) showed relatively more connections with the Visual A and Dorsal Attention A networks, while anterior scene regions (aPPA, RSC) had more connections with the Visual B, Control C, Default A and Default C networks. Meanwhile, Figure 5e shows the tractography results between the core scene regions and subcortical structures, and contrasts between regions are shown in Figure S13e. As noted in the main ROI analyses, anterior scene regions (aPPA, RSC) showed more connections with the hippocampus. In addition, the posterior PPA and OPA showed relatively high connection probabilities with the putamen.

Finally, to summarise the connections through the white matter, we examined the structural connectivity between each of the core scene regions and major white matter tracts throughout the brain. We used the XTRACT toolbox (Warrington et al., 2020) to generate individualised reconstructions of 42 major white matter tracts. We then ran a whole‐brain tractography analysis, seeding from each surface vertex along the white/grey‐matter boundary. Next, we produced whole‐brain connectivity blueprints by measuring the similarity in the white matter connectivity patterns between each tract and each vertex—the resulting statistical maps are proportional to the probability of a given tract terminating at each location along the white/grey‐matter boundary. Group‐average heat maps for each tract are illustrated in Figure S18. We averaged these blueprints over vertices within each of the core scene regions to estimate the degree of connectivity between each ROI and each tract (Figure 6e). This revealed patterns of connectivity following both posterior/anterior and ventral/dorsal divisions. The vertical occipital fasciculus (VOF) showed a high probability of terminating within posterior scene regions (OPA, pPPA), while the dorsal and temporal subsections of the cingulum bundle (CBD, CBT) were more likely to terminate within anterior scene regions (aPPA, RSC). The inferior fronto‐occipital fasciculus (IFOF) and optic radiation (OR) both showed high connection probabilities with all scene regions, with the IFOF additionally showing a bias towards the ventrally located PPA. The inferior longitudinal fasciculus (ILF) also showed preferential connectivity with the PPA, while the middle longitudinal fasciculus (MdLF) appeared biased towards the dorsally located RSC and OPA. Finally, all regions (but especially the RSC) indicated connectivity with the forceps major (FMA), supporting interhemispheric connectivity via the splenium. Taken together, the structural connectivity profile of the scene network indicates some distinctions between posterior and anterior regions, but also between ventral and dorsal regions which were not so evident in the functional connectivity profiles.

### 3.3 Comparison between connectivity measures

**FIGURE 9:**
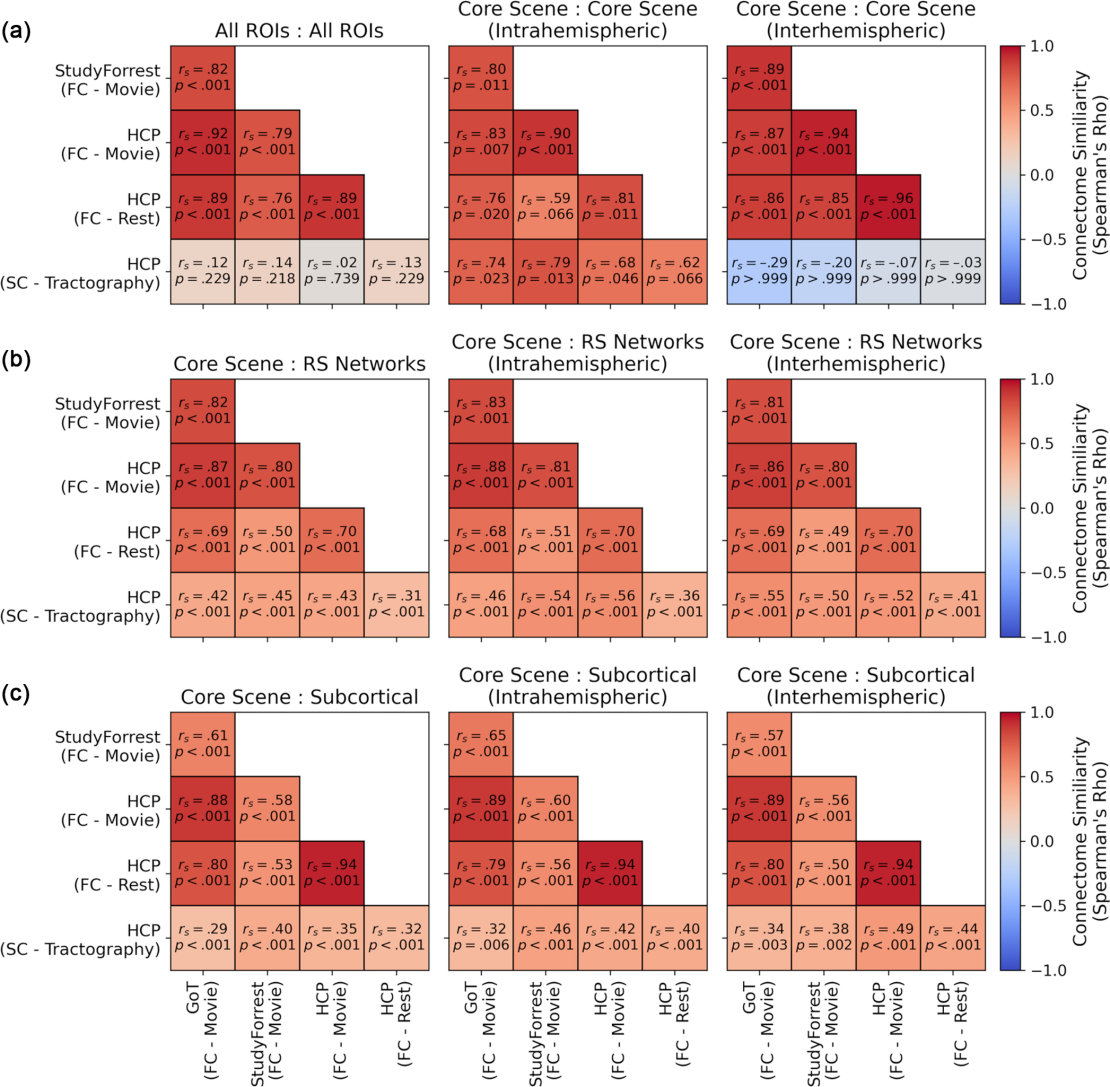
Similarity between connectivity estimates. (a) Connectivity matrices between early visual and core and extended scene regions (cf. Figures 1a and 6a) were correlated between the functional connectivity estimates in each dataset and the structural connectivity estimates from the HCP dataset. Additional analyses restricted the comparisons to just intrahemispheric or interhemispheric connections between core scene regions. Further similarity analyses compared connectivity matrices (b) between core scene regions and 17 resting‐state networks (cf. Figure 4) and (c) between core scene regions and subcortical structures (cf. Figure 5), either including all connections or just intrahemispheric or interhemispheric connections. Annotations indicate correlation and *p* values following a Holm‐Bonferroni correction for multiple comparisons.

Finally, we correlated the functional (Game of Thrones, StudyForrest, HCP movie‐watching, HCP resting‐state) and structural connectivity measures between datasets. Figure 9a shows the comparison between connectivity estimates for early visual and core and extended scene regions (cf. Figures 1a and 6a). A high degree of consistency was observed between all functional connectivity estimates, including between movie‐watching and resting‐state HCP datasets, however functional and structural connectivity estimates showed relatively poor correspondence. The similarity could be confounded by the tendency for connectivity to be stronger within than between networks—to test this we further measured the similarity for intrahemispheric or interhemispheric connections between the core scene regions only. In both cases, the functional connectivity estimates remained highly correlated. Interestingly, a high degree of similarity was also evident between the functional and structural connectivity for the intrahemispheric (though not interhemispheric) connections.

Comparisons of connectivity estimates between core scene regions and resting‐state networks are shown in Figure 9b (cf. Figure 4) and comparisons of estimates between core scene regions and subcortical structures are shown in Figure 9c (cf. Figure 5). We performed these analyses including all, just intrahemispheric, or just interhemispheric connections. In all cases, a high degree of similarity was observed between functional connectivity estimates, and moderate correlations were also found between functional and structural connectivity estimates—including for both intrahemispheric and interhemispheric connections. Thus, functional connectivity estimates were robust across datasets and between movie‐watching and rest. Although the structural connectivity did not predict the functional connectivity well for the full connectome of main ROIs or interhemispheric connections within the scene network, a greater degree of correspondence was observed for intrahemispheric scene connections and for connections with the resting‐state networks and subcortical structures.

## 4 DISCUSSION

In this study we mapped the functional and structural connectivity profile of the scene processing network during natural viewing and at rest. The analysis of functional connectivity revealed a bias in connectivity between the posterior (OPA and pPPA) and anterior (aPPA and RSC) core scene regions. The anterior scene network connected more strongly with default mode and frontoparietal control networks and the hippocampus, while the posterior scene network connected more strongly with early visual cortices and the dorsal attention network. A similar pattern was evident in the analysis of structural connectivity, again highlighting distinctions between posterior and anterior regions, but also distinctions between ventral and dorsal regions.

We began by measuring functional connectivity, during movie‐watching and at rest, between the core and extended scene regions (Baldassano et al., 2016; Epstein & Baker, 2019) and early visual regions. In line with previous resting‐state studies (Baldassano et al., 2013; Boccia et al., 2017; Nasr et al., 2013; Silson et al., 2016; Tullo et al., 2023), all datasets indicated a bias between posterior and anterior scene networks. Posterior scene regions including the OPA and posterior PPA showed preferential connectivity with early visual regions, consistent with a role in processing egocentric visual features of scenes (Baldassano et al., 2016). Connectivity between the OPA and PPA is consistent with evidence that applying TMS to the OPA disrupts functional responses in the posterior PPA (Groen et al., 2021; Rafique et al., 2015). Meanwhile, anterior scene regions including the RSC and anterior PPA connected preferentially with extended scene regions including the hippocampus and cIPL, consistent with a role in higher‐level aspects of scene processing such as memory and navigation. These results are also consistent with recent studies of effective functional connectivity indicating a ventromedial stream including posterior stages connecting early visual and parahippocampal cortices, followed by anterior stages further connecting parahippocampal and medial temporal lobe regions (Rolls, Deco, Huang, & Feng, 2023; Rolls, Deco, Zhang, & Feng, 2023). Nevertheless, we also observed strong connectivity between the core scene regions themselves. Thus, our results argue for a bias rather than a hard division between posterior and anterior networks. Interestingly, there was a clearer distinction between anterior and posterior networks in the resting‐state compared to the movie‐watching datasets. Previous proposals of a hard division between networks (Baldassano et al., 2016) have relied on studies measuring functional connectivity at rest. These studies may, therefore, have estimated a greater distinction between the networks than would have been obtained during naturalistic viewing.

We next measured functional connectivity between the scene regions and the rest of the brain using seed‐based analyses and by examining connectivity with cortical resting‐state networks (Yeo et al., 2011) and subcortical structures. Again, we asked whether there were differences in the patterns of connectivity between regions in the anterior and posterior components of the scene network. The posterior scene network connected preferentially with component A of the visual network, which includes ventral/lateral occipital and temporal cortices. This is consistent with the proposed role of these regions in processing egocentric visual features of the scene (Baldassano et al., 2016). However, we also observed preferential connectivity with the dorsal attention network (Fox et al., 2006; Vossel et al., 2014), which includes lateral occipital and posterior parietal cortices. These regions are implicated in visuospatial attentional control, suggesting a role within the dorsal visual stream. Consistent with this, Malcolm et al. (2018) report that applying TMS to the OPA biases eye movements during scene viewing.

In contrast, the anterior scene network showed preferential connectivity with regions of the default mode network (Greicius et al., 2003; Raichle et al., 2001), including lateral parietal regions (including the inferior parietal lobule), medial parietal regions (including the precuneus and posterior cingulate cortex) and medial prefrontal cortices. Similar patterns of connectivity for RSC and posterior cingulate seeds have been reported previously (Margulies et al., 2009). These regions overlap with the parietal‐hippocampal component of the default mode network (Vincent et al., 2006), which also includes parahippocampal and retrosplenial cortices, and is implicated in memory recall. This is consistent with a proposed network of place memory areas located anterior to the core scene network which are implicated in the representation of familiar scenes (Silson et al., 2019; Steel et al., 2021, 2023, 2024). Functional connectivity measured during movie watching indicates that this network is linked to, but nevertheless functionally distinct from, the core scene network (Steel et al., 2021). Anterior scene regions also displayed preferential connectivity with component C of the frontoparietal control network, including the superior precuneus and posterior cingulate regions, which is implicated in cognitive control (Dosenbach et al., 2007; Vincent et al., 2008). These results support a role for the anterior scene network in higher‐level aspects of scene processing such as navigation and memory (Baldassano et al., 2016). Within subcortical structures, anterior scene regions showed preferential connectivity with the hippocampus and, less consistently, the thalamus, while both anterior and posterior scene networks showed spatially distinct clusters of preferential connectivity within the cerebellum. The connectivity between anterior scene regions and the hippocampus has been related to the retrieval of imagined places (Tullo et al., 2023). Indeed, imagery and recall of familiar environments encompasses many of the extended scene regions implicated in connectivity with the anterior scene network (Boccia et al., 2015), consistent with proposed mnemonic functions of this network.

Seed connectivity analyses generally highlighted more extensive functional connectivity throughout the brain during rest than in movie‐watching. This is consistent with previous literature (Betti et al., 2013; Lynch et al., 2018; Wang et al., 2017), and likely reflects movie‐watching disrupting the spontaneous intrinsic brain activity that underpins resting‐state functional connectivity. Nevertheless, the overall pattern of connectivity appeared consistent between movie‐watching and rest. Movie‐watching can offer advantages over resting‐state paradigms, such as improved prediction of brain function (Gal et al., 2022; Knapen, 2021) and human behaviour (Finn & Bandettini, 2021), and the possibility of examining intersubject correlations (Hasson et al., 2004; Simony et al., 2016). Thus, movie‐watching may provide a promising paradigm for future investigations of connectivity within the scene network.

Both region of interest and seed‐based analyses highlighted clear patterns of interhemispheric functional connectivity. For instance, region of interest analyses indicated strong connectivity between homotopic regions in each hemisphere (e.g., between left and right OPA). Indeed, homotopic regions paired together most immediately within the hierarchical clustering and appeared proximal to each other within the multidimensional scaling solution. The relative pattern of connectivity between regions also appeared similar within and between hemispheres, for instance the biases between anterior and posterior regions were evident in both intrahemispheric and interhemispheric connections. Furthermore, seed‐based analyses highlighted extensive bilateral patterns of functional connectivity for each scene region. Thus, while previous models of the scene network have emphasised the role of intrahemispheric connections between regions (Baldassano et al., 2016), our results additionally underscore the importance of interhemispheric connectivity.

We also examined the structural connectivity between early visual and core and extended scene regions using tractography analyses. We found good correspondence between functional and structural estimates of intrahemispheric connections between the core scene regions. For instance, the RSC and the anterior PPA appeared highly connected with the hippocampus in both structural and functional connectivity. This is consistent with neuronal tracing studies of monkeys showing a parieto‐medial pathway connecting RSC and posterior cingulate cortices with medial temporal regions (Kravitz et al., 2011). However, structural connectivity was more heavily influenced by the anatomy than functional connectivity. For instance, there were a high proportion of streamlines between the OPA and cIPL due to their anatomical proximity, despite these regions being minimally functionally connected. We also observed relatively few interhemispheric connections between regions, although the intrahemispheric pattern of connectivity appeared similar in each hemisphere. Consequently, the structural connectivity between regions displayed relatively poor correspondence with the functional connectivity for the connectome as a whole. These discrepancies may reflect limitations of the diffusion tractography technique, or that the functional connectivity may reflect more indirect connections. However, we did find good correspondence between the functional and structural connectivity of the scene network when the analysis was restricted to intrahemispheric connections. Contrary to Kim et al. (2006), we found little evidence of structural connections between PPA and early visual cortex—this may reflect differences between the tractography algorithms employed (probabilistic versus deterministic) or that the HCP provides a much larger sample size.

Structural connectivity between the core scene regions and the rest of the brain revealed both similarities and differences with the functional connectivity. Whole‐brain tractography analyses primarily highlighted a ventral/dorsal distinction, with the PPA displaying higher connection probabilities along the temporal lobe, while the OPA and RSC were more likely to show parietal, frontal and interhemispheric connections. However, grey‐to‐grey matter structural connections between the core scene regions and both cortical resting‐state networks and subcortical structures showed better correspondence with the functional connectivity estimates. Connectivity with the resting‐state networks (Yeo et al., 2011) indicated a higher proportion of connections from posterior scene regions to the Visual A and Dorsal Attention A networks, and a higher proportion of connections from anterior scene regions to the Visual B, Control C and Default‐Mode A and C networks. Connectivity with the subcortical regions revealed a high proportion of connections from the PPA and RSC to the hippocampus, consistent with the functional connectivity. A high proportion of connections were also observed from the posterior PPA and OPA to the putamen. Thus, structural connectivity estimates highlighted some biases between posterior versus anterior regions consistent with the functional connectivity, but also biases between ventral and dorsal regions.

We also examined the structural connectivity between each core scene region and major white matter tracts throughout the brain (Warrington et al., 2020). Some tracts again highlighted biases between posterior and anterior scene regions. For instance, the RSC and anterior PPA were connected with the cingulum bundle, particularly the temporal subsection which connects posterior cingulate and medial temporal regions, and to a lesser extent the dorsal subsection which further projects rostrally along the cingulate cortex (Heilbronner & Haber, 2014). These tracts are consistent with the structural parieto‐medial pathway proposed in the monkey literature (Kravitz et al., 2011). Meanwhile, the OPA and posterior PPA connected with the vertical occipital fasciculus, which has been suggested to support communication between ventral and dorsal visual streams (Takemura et al., 2016). Other tracts highlighted distinctions between ventral and dorsal scene regions. The ventrally‐located anterior and posterior PPA connected with the inferior longitudinal fasciculus (Catani et al., 2003), which runs laterally along the temporal lobe connecting occipital and anterior temporal regions. All scene regions, though especially the anterior and posterior PPA, also connected with the inferior fronto‐occipital fasciculus (Catani et al., 2002), which runs medially along the temporal lobe connecting occipital and prefrontal cortices. These two fasciculi are thought to support many visual functions of the ventral stream, including the processing of faces, objects and places (Herbet et al., 2018; Rokem et al., 2017). Meanwhile, the dorsally located OPA and RSC connected with the middle longitudinal fasciculus, which connects dorsal anterior temporal regions with dorsal and lateral occipital regions (Makris et al., 2009; Maldonado et al., 2013). Finally, all scene regions (and especially the RSC) connected to the forceps major, supporting interhemispheric connections via the splenium. This tract has been implicated in supporting interhemispheric connectivity between homotopic visual regions (Dougherty et al., 2005; Saenz & Fine, 2010). A key discrepancy between the functional and structural connectivity is the lack of interhemispheric connections between regions in the latter. It is therefore likely that interhemispheric connectivity is supported by white matter tracts passing through the splenium, but the tractography analysis is not sufficiently sensitive to completely trace the tracts between regions in each hemisphere.

In conclusion, we describe the functional and structural connectivity profiles of the scene processing network. Functional connectivity estimates supported a bias between posterior and anterior scene networks which converge in the PPA. The posterior network was more strongly connected with early visual and dorsal attention networks, suggesting a role in processing egocentric visual features of scenes and also a function within the dorsal visual stream. The anterior network was more strongly connected with default mode and cognitive control networks, implying a role in higher‐level aspects of scene perception such as memory, recognition and navigation. Functional connectivity profiles also underscored the importance of interhemispheric connectivity and were robust across resting‐state and movie‐watching datasets. Structural connectivity profiles generally showed good correspondence with the functional connectivity, and highlighted some biases between posterior and anterior regions, but also between ventral and dorsal regions. Taken together, these findings provide a map of the connectivity of the scene network, informing possible roles for scene‐selective regions and their network interactions in brain function and human behaviour.

## AUTHOR CONTRIBUTIONS

Both authors conceived and developed the experiments. D.M.W. performed the data analysis under the supervision of T.J.A. Both authors contributed to the writing of the manuscript and approved the final version for submission.

## CONFLICT OF INTEREST STATEMENT

There is no conflict of interest.

## Supporting information


**Data S1:** Supporting information.

**Video S1:** 3D renderings of seed‐based tractography from core scene regions. Statistical overlays illustrate FWER‐corrected TFCE *p*‐values for one‐sample tests of connection probabilities against zero over subjects.

**Video S2:** 3D renderings of contrasts of seed‐based tractography between left hemisphere core scene regions. Statistical overlays illustrate FWER‐corrected TFCE *p*‐values for paired‐sample tests of connection probabilities between seed regions over subjects.

**Video S3:** 3D renderings of contrasts of seed‐based tractography between right hemisphere core scene regions. Statistical overlays illustrate FWER‐corrected TFCE *p*‐values for paired‐sample tests of connection probabilities between seed regions over subjects.

## Data Availability

The Game of Thrones dataset is available on OpenNeuro (https://openneuro.org/datasets/ds004848). The StudyForrest (https://www.studyforrest.org/) and Human Connectome Project (https://db.humanconnectome.org) datasets were obtained from already publicly available repositories.

## References

[hbm26628-bib-0001] Alberton, B. A. V. , Nichols, T. E. , Gamba, H. R. , & Winkler, A. M. (2020). Multiple testing correction over contrasts for brain imaging. NeuroImage, 216, 116760. 32201328 10.1016/j.neuroimage.2020.116760 PMC8191638

[hbm26628-bib-0002] Andersson, J. L. R. , Jenkinson, M. , & Smith, S. (2010). Non‐linear registration, aka spatial normalization (Publication No. TR07JA2). FMRIB Centre.

[hbm26628-bib-0003] Baldassano, C. , Beck, D. M. , & Fei‐Fei, L. (2013). Differential connectivity within the Parahippocampal Place Area. NeuroImage, 75, 228–237. 23507385 10.1016/j.neuroimage.2013.02.073 PMC3683120

[hbm26628-bib-0004] Baldassano, C. , Esteva, A. , Fei‐Fei, L. , & Beck, D. M. (2016). Two distinct scene‐processing networks connecting vision and memory. eNeuro, 3(5), 1–14. 10.1523/ENEURO.0178-16.2016 PMC5075944 27822493

[hbm26628-bib-0005] Beckmann, C. F. , & Smith, S. M. (2004). Probabilistic independent component analysis for functional magnetic resonance imaging. IEEE Transactions on Medical Imaging, 23(2), 137–152. 14964560 10.1109/TMI.2003.822821

[hbm26628-bib-0006] Behzadi, Y. , Restom, K. , Liau, J. , & Liu, T. T. (2007). A component based noise correction method (CompCor) for BOLD and perfusion based fMRI. NeuroImage, 37(1), 90–101. 17560126 10.1016/j.neuroimage.2007.04.042 PMC2214855

[hbm26628-bib-0007] Benson, N. C. , Butt, O. H. , Brainard, D. H. , & Aguirre, G. K. (2014). Correction of distortion in flattened representations of the cortical surface allows prediction of V1‐V3 functional organization from anatomy. PLoS Computational Biology, 10(3), e1003538. 24676149 10.1371/journal.pcbi.1003538 PMC3967932

[hbm26628-bib-0008] Benson, N. C. , Jamison, K. W. , Arcaro, M. J. , Vu, A. T. , Glasser, M. F. , Coalson, T. S. , Van Essen, D. C. , Yacoub, E. , Ugurbil, K. , Winawer, J. , & Kay, K. (2018). The Human Connectome Project 7 Tesla retinotopy dataset: Description and population receptive field analysis. Journal of Vision, 18(13), 23. 10.1167/18.13.23 PMC6314247 30593068

[hbm26628-bib-0009] Benson, N. C. , & Winawer, J. (2018). Bayesian analysis of retinotopic maps. eLife, 7, 1–29. 10.7554/eLife.40224 PMC6340702 30520736

[hbm26628-bib-0010] Betti, V. , Della Penna, S. , de Pasquale, F. , Mantini, D. , Marzetti, L. , Romani, G. L. , & Corbetta, M. (2013). Natural scenes viewing alters the dynamics of functional connectivity in the human brain. Neuron, 79(4), 782–797. 23891400 10.1016/j.neuron.2013.06.022 PMC3893318

[hbm26628-bib-0011] Boccia, M. , Piccardi, L. , Palermo, L. , Nemmi, F. , Sulpizio, V. , Galati, G. , & Guariglia, C. (2015). A penny for your thoughts! Patterns of fMRI activity reveal the content and the spatial topography of visual mental images. Human Brain Mapping, 36(3), 945–958. 25359694 10.1002/hbm.22678 PMC6869538

[hbm26628-bib-0012] Boccia, M. , Sulpizio, V. , Nemmi, F. , Guariglia, C. , & Galati, G. (2017). Direct and indirect parieto‐medial temporal pathways for spatial navigation in humans: Evidence from resting‐state functional connectivity. Brain Structure and Function, 222(4), 1945–1957. 27704218 10.1007/s00429-016-1318-6

[hbm26628-bib-0013] Caspers, S. , Geyer, S. , Schleicher, A. , Mohlberg, H. , Amunts, K. , & Zilles, K. (2006). The human inferior parietal cortex: Cytoarchitectonic parcellation and interindividual variability. NeuroImage, 33(2), 430–448. 16949304 10.1016/j.neuroimage.2006.06.054

[hbm26628-bib-0014] Catani, M. , Howard, R. J. , Pajevic, S. , & Jones, D. K. (2002). Virtual in vivo interactive dissection of white matter fasciculi in the human brain. NeuroImage, 17(1), 77–94. 12482069 10.1006/nimg.2002.1136

[hbm26628-bib-0015] Catani, M. , Jones, D. K. , Donato, R. , & Ffytche, D. H. (2003). Occipito‐temporal connections in the human brain. Brain, 126(9), 2093–2107. 12821517 10.1093/brain/awg203

[hbm26628-bib-0016] Cox, R. W. (1996). AFNI: Software for analysis and visualization of functional magnetic resonance neuroimages. Computers and Biomedical Research, 29(29), 162–173. 8812068 10.1006/cbmr.1996.0014

[hbm26628-bib-0017] Cutting, J. E. , Brunick, K. L. , & Candan, A. (2012). Perceiving event dynamics and parsing Hollywood films. Journal of Experimental Psychology: Human Perception and Performance, 38(6), 1476–1490. 22449126 10.1037/a0027737

[hbm26628-bib-0018] Dale, A. M. , Fischl, B. , & Sereno, M. I. (1999). Cortical surface‐based analysis I. Segmentation and surface reconstruction. NeuroImage, 9(2), 179–194. 9931268 10.1006/nimg.1998.0395

[hbm26628-bib-0019] Dilks, D. D. , Julian, J. B. , Paunov, A. M. , & Kanwisher, N. (2013). The occipital place area is causally and selectively involved in scene perception. Journal of Neuroscience, 33(4), 1331–1336. 23345209 10.1523/JNEUROSCI.4081-12.2013 PMC3711611

[hbm26628-bib-0020] Dosenbach, N. U. F. , Fair, D. A. , Miezin, F. M. , Cohen, A. L. , Wenger, K. K. , Dosenbach, R. A. T. , Fox, M. D. , Snyder, A. Z. , Vincent, J. L. , Raichle, M. E. , Schlaggar, B. L. , & Petersen, S. E. (2007). Distinct brain networks for adaptive and stable task control in humans. Proceedings of the National Academy of Sciences, 104(26), 11073–11078. 10.1073/pnas.0704320104 PMC1904171 17576922

[hbm26628-bib-0021] Dougherty, R. F. , Ben‐Shachar, M. , Bammer, R. , Brewer, A. A. , & Wandell, B. A. (2005). Functional organization of human occipital‐callosal fiber tracts. Proceedings of the National Academy of Sciences, 102(20), 7350–7355. 10.1073/pnas.0500003102 PMC1129102 15883384

[hbm26628-bib-0022] Eickhoff, S. B. , Stephan, K. E. , Mohlberg, H. , Grefkes, C. , Fink, G. R. , Amunts, K. , & Zilles, K. (2005). A new SPM toolbox for combining probabilistic cytoarchitectonic maps and functional imaging data. NeuroImage, 25(4), 1325–1335. 15850749 10.1016/j.neuroimage.2004.12.034

[hbm26628-bib-0023] Epstein, R. A. , & Baker, C. I. (2019). Scene perception in the human brain. Annual Review of Vision Science, 5(1), 373–397. 10.1146/annurev-vision-091718-014809 PMC6989029 31226012

[hbm26628-bib-0024] Epstein, R. A. , & Kanwisher, N. (1998). A cortical representation of the local visual environment. Nature, 392(6676), 598–601. 9560155 10.1038/33402

[hbm26628-bib-0025] Finn, E. S. (2021). Is it time to put rest to rest? Trends in Cognitive Sciences, 25(12), 1021–1032. 34625348 10.1016/j.tics.2021.09.005 PMC8585722

[hbm26628-bib-0026] Finn, E. S. , & Bandettini, P. A. (2021). Movie‐watching outperforms rest for functional connectivity‐based prediction of behavior. NeuroImage, 235, 117963. 33813007 10.1016/j.neuroimage.2021.117963 PMC8204673

[hbm26628-bib-0027] Fischl, B. , Salat, D. H. , Busa, E. , Albert, M. , Dieterich, M. , Haselgrove, C. , van der Kouwe, A. , Killiany, R. , Kennedy, D. , Klaveness, S. , Montillo, A. , Makris, N. , Rosen, B. , & Dale, A. M. (2002). Whole brain segmentation: Automated labeling of neuroanatomical structures in the human brain. Neuron, 33(3), 341–355. 11832223 10.1016/s0896-6273(02)00569-x

[hbm26628-bib-0028] Fox, M. D. , Corbetta, M. , Snyder, A. Z. , Vincent, J. L. , & Raichle, M. E. (2006). Spontaneous neuronal activity distinguishes human dorsal and ventral attention systems. Proceedings of the National Academy of Sciences, 103(26), 10046–10051. 10.1073/pnas.0604187103 PMC1480402 16788060

[hbm26628-bib-0029] Gal, S. , Coldham, Y. , Tik, N. , Bernstein‐Eliav, M. , & Tavor, I. (2022). Act natural: Functional connectivity from naturalistic stimuli fMRI outperforms resting‐state in predicting brain activity. NeuroImage, 258, 119359. 35680054 10.1016/j.neuroimage.2022.119359

[hbm26628-bib-0030] Glasser, M. F. , Sotiropoulos, S. N. , Wilson, J. A. , Coalson, T. S. , Fischl, B. , Andersson, J. L. , Xu, J. , Jbabdi, S. , Webster, M. , Polimeni, J. R. , Van Essen, D. C. , & Jenkinson, M. (2013). The minimal preprocessing pipelines for the Human Connectome Project. NeuroImage, 80, 105–124. 23668970 10.1016/j.neuroimage.2013.04.127 PMC3720813

[hbm26628-bib-0031] Greicius, M. D. , Krasnow, B. , Reiss, A. L. , & Menon, V. (2003). Functional connectivity in the resting brain: A network analysis of the default mode hypothesis. Proceedings of the National Academy of Sciences, 100(1), 253–258. 10.1073/pnas.0135058100 PMC140943 12506194

[hbm26628-bib-0032] Greicius, M. D. , Supekar, K. , Menon, V. , & Dougherty, R. F. (2009). Resting‐state functional connectivity reflects structural connectivity in the default mode network. Cerebral Cortex, 19(1), 72–78. 18403396 10.1093/cercor/bhn059 PMC2605172

[hbm26628-bib-0033] Greve, D. N. , & Fischl, B. (2009). Accurate and robust brain image alignment using boundary‐based registration. NeuroImage, 48(1), 63–72. 19573611 10.1016/j.neuroimage.2009.06.060 PMC2733527

[hbm26628-bib-0034] Groen, I. I. A. , Silson, E. H. , Pitcher, D. , & Baker, C. I. (2021). Theta‐burst TMS of lateral occipital cortex reduces BOLD responses across category‐selective areas in ventral temporal cortex. NeuroImage, 230, 117790. 33497776 10.1016/j.neuroimage.2021.117790 PMC8094793

[hbm26628-bib-0035] Hanke, M. , Adelhöfer, N. , Kottke, D. , Iacovella, V. , Sengupta, A. , Kaule, F. R. , Nigbur, R. , Waite, A. Q. , Baumgartner, F. , & Stadler, J. (2016). A studyforrest extension, simultaneous fMRI and eye gaze recordings during prolonged natural stimulation. Scientific Data, 3(1), 160092. 27779621 10.1038/sdata.2016.92 PMC5079121

[hbm26628-bib-0036] Hanke, M. , Baumgartner, F. J. , Ibe, P. , Kaule, F. R. , Pollmann, S. , Speck, O. , Zinke, W. , & Stadler, J. (2014). A high‐resolution 7‐tesla fMRI dataset from complex natural stimulation with an audio movie. Scientific Data, 1(1), 140003. 25977761 10.1038/sdata.2014.3 PMC4322572

[hbm26628-bib-0037] Hasson, U. , Nir, Y. , Levy, I. , Fuhrmann, G. , & Malach, R. (2004). Intersubject synchronization of cortical activity during natural vision. Science, 303(5664), 1634–1640. 15016991 10.1126/science.1089506

[hbm26628-bib-0038] Heilbronner, S. R. , & Haber, S. N. (2014). Frontal cortical and subcortical projections provide a basis for segmenting the cingulum bundle: Implications for neuroimaging and psychiatric disorders. Journal of Neuroscience, 34(30), 10041–10054. 25057206 10.1523/JNEUROSCI.5459-13.2014 PMC4107396

[hbm26628-bib-0039] Herbet, G. , Zemmoura, I. , & Duffau, H. (2018). Functional anatomy of the inferior longitudinal fasciculus: From historical reports to current hypotheses. Frontiers in Neuroanatomy, 12, 1–15. 30283306 10.3389/fnana.2018.00077 PMC6156142

[hbm26628-bib-0040] Jenkinson, M. , Bannister, P. , Brady, M. , & Smith, S. (2002). Improved optimization for the robust and accurate linear registration and motion correction of brain images. NeuroImage, 17(2), 825–841. 12377157 10.1016/s1053-8119(02)91132-8

[hbm26628-bib-0041] Jenkinson, M. , Beckmann, C. F. , Behrens, T. E. J. , Woolrich, M. W. , & Smith, S. M. (2012). FSL. NeuroImage, 62(2), 782–790. 21979382 10.1016/j.neuroimage.2011.09.015

[hbm26628-bib-0042] Julian, J. B. , Keinath, A. T. , Marchette, S. A. , & Epstein, R. A. (2018). The neurocognitive basis of spatial reorientation. Current Biology, 28(17), R1059–R1073. 30205055 10.1016/j.cub.2018.04.057 PMC6161705

[hbm26628-bib-0043] Kim, M. , Ducros, M. , Carlson, T. , Ronen, I. , He, S. , Ugurbil, K. , & Kim, D. S. (2006). Anatomical correlates of the functional organization in the human occipitotemporal cortex. Magnetic Resonance Imaging, 24(5), 583–590. 16735179 10.1016/j.mri.2005.12.005

[hbm26628-bib-0044] Knapen, T. (2021). Topographic connectivity reveals task‐dependent retinotopic processing throughout the human brain. Proceedings of the National Academy of Sciences, 118(2), e2017032118. 10.1073/pnas.2017032118 PMC7812773 33372144

[hbm26628-bib-0045] Kobayashi, Y. , & Amaral, D. G. (2003). Macaque monkey retrosplenial cortex: II. Cortical afferents. The Journal of Comparative Neurology, 466(1), 48–79. 14515240 10.1002/cne.10883

[hbm26628-bib-0046] Kobayashi, Y. , & Amaral, D. G. (2007). Macaque monkey retrosplenial cortex: III. Cortical efferents. The Journal of Comparative Neurology, 502(5), 810–833. 17436282 10.1002/cne.21346

[hbm26628-bib-0047] Kravitz, D. J. , Saleem, K. S. , Baker, C. I. , & Mishkin, M. (2011). A new neural framework for visuospatial processing. Nature Reviews Neuroscience, 12(4), 217–230. 21415848 10.1038/nrn3008 PMC3388718

[hbm26628-bib-0048] Langner, O. , Dotsch, R. , Bijlstra, G. , Wigboldus, D. H. J. , Hawk, S. T. , & van Knippenberg, A. (2010). Presentation and validation of the Radboud Faces Database. Cognition & Emotion, 24(8), 1377–1388.

[hbm26628-bib-0049] Lynch, L. K. , Lu, K. , Wen, H. , Zhang, Y. , Saykin, A. J. , & Liu, Z. (2018). Task‐evoked functional connectivity does not explain functional connectivity differences between rest and task conditions. Human Brain Mapping, 39(12), 4939–4948. 30144210 10.1002/hbm.24335 PMC6397020

[hbm26628-bib-0050] Maguire, E. (2001). The retrosplenial contribution to human navigation: A review of lesion and neuroimaging findings. Scandinavian Journal of Psychology, 42(3), 225–238. 11501737 10.1111/1467-9450.00233

[hbm26628-bib-0051] Makris, N. , Papadimitriou, G. M. , Kaiser, J. R. , Sorg, S. , Kennedy, D. N. , & Pandya, D. N. (2009). Delineation of the middle longitudinal fascicle in humans: A quantitative, in vivo, DT‐MRI study. Cerebral Cortex, 19(4), 777–785. 18669591 10.1093/cercor/bhn124 PMC2651473

[hbm26628-bib-0052] Malcolm, G. L. , Silson, E. H. , Henry, J. R. , & Baker, C. I. (2018). Transcranial magnetic stimulation to the occipital place area biases gaze during scene viewing. Frontiers in Human Neuroscience, 12, 1–13. 29867413 10.3389/fnhum.2018.00189 PMC5953332

[hbm26628-bib-0053] Maldonado, I. L. , De Champfleur, N. M. , Velut, S. , Destrieux, C. , Zemmoura, I. , & Duffau, H. (2013). Evidence of a middle longitudinal fasciculus in the human brain from fiber dissection. Journal of Anatomy, 223(1), 38–45. 23621438 10.1111/joa.12055 PMC3798102

[hbm26628-bib-0054] Margulies, D. S. , Vincent, J. L. , Kelly, C. , Lohmann, G. , Uddin, L. Q. , Biswal, B. B. , Villringer, A. , Castellanos, F. X. , Milham, M. P. , & Petrides, M. (2009). Precuneus shares intrinsic functional architecture in humans and monkeys. Proceedings of the National Academy of Sciences, 106(47), 20069–20074. 10.1073/pnas.0905314106 PMC2775700 19903877

[hbm26628-bib-0055] Nasr, S. , Devaney, K. J. , & Tootell, R. B. H. (2013). Spatial encoding and underlying circuitry in scene‐selective cortex. NeuroImage, 83, 892–900. 23872156 10.1016/j.neuroimage.2013.07.030 PMC3815999

[hbm26628-bib-0056] Noad, K. , Watson, D. M. , & Andrews, T. J. (n.d.). Natural viewing reveals an extended network of regions for familiar faces that is disrupted in developmental prosopagnosia. (In review).

[hbm26628-bib-0057] O'Keefe, J. , & Nadel, L. (1978). The hippocampus as a cognitive map. Oxford University Press.

[hbm26628-bib-0058] Pedregosa, F. , Varoquaux, G. , Gramfort, A. , Michel, V. , Thirion, B. , Grisel, O. , Blondel, M. , Prettenhofer, P. , Weiss, R. , Dubourg, V. , Vanderplas, J. , Passos, A. , Cournapeau, D. , Brucher, M. , Perrot, M. , & Duchesnay, E. (2011). Scikit‐learn: Machine learning in python. Journal of Machine Learning Research, 12, 2825–2830.

[hbm26628-bib-0059] Peirce, J. , Gray, J. R. , Simpson, S. , MacAskill, M. , Höchenberger, R. , Sogo, H. , Kastman, E. , & Lindeløv, J. K. (2019). PsychoPy2: Experiments in behavior made easy. Behavior Research Methods, 3, 195–203. 10.3758/s13428-018-01193-y PMC6420413 30734206

[hbm26628-bib-0060] Pruim, R. H. R. , Mennes, M. , van Rooij, D. , Llera, A. , Buitelaar, J. K. , & Beckmann, C. F. (2015). ICA‐AROMA: A robust ICA‐based strategy for removing motion artifacts from fMRI data. NeuroImage, 112, 267–277. 25770991 10.1016/j.neuroimage.2015.02.064

[hbm26628-bib-0061] Rafique, S. A. , Solomon‐Harris, L. M. , & Steeves, J. K. E. (2015). TMS to object cortex affects both object and scene remote networks while TMS to scene cortex only affects scene networks. Neuropsychologia, 79, 86–96. 26511624 10.1016/j.neuropsychologia.2015.10.027

[hbm26628-bib-0062] Raichle, M. E. , MacLeod, A. M. , Snyder, A. Z. , Powers, W. J. , Gusnard, D. A. , & Shulman, G. L. (2001). A default mode of brain function. Proceedings of the National Academy of Sciences, 98(2), 676–682. 10.1073/pnas.98.2.676 PMC14647 11209064

[hbm26628-bib-0063] Robinson, E. C. , Garcia, K. , Glasser, M. F. , Chen, Z. , Coalson, T. S. , Makropoulos, A. , Bozek, J. , Wright, R. , Schuh, A. , Webster, M. , Hutter, J. , Price, A. , Grande, L. C. , Hughes, E. , Tusor, N. , Bayly, P. V. , Van Essen, D. C. , Smith, S. M. , Edwards, A. D. , … Rueckert, D. (2018). Multimodal surface matching with higher‐order smoothness constraints. NeuroImage, 167, 453–465. 29100940 10.1016/j.neuroimage.2017.10.037 PMC5991912

[hbm26628-bib-0064] Robinson, E. C. , Jbabdi, S. , Glasser, M. F. , Andersson, J. , Burgess, G. C. , Harms, M. P. , Smith, S. M. , Van Essen, D. C. , & Jenkinson, M. (2014). MSM: A new flexible framework for multimodal surface matching. NeuroImage, 100, 414–426. 24939340 10.1016/j.neuroimage.2014.05.069 PMC4190319

[hbm26628-bib-0065] Rokem, A. , Takemura, H. , Bock, A. S. , Scherf, K. S. , Behrmann, M. , Wandell, B. A. , Fine, I. , Bridge, H. , & Pestilli, F. (2017). The visual white matter: The application of diffusion MRI and fiber tractography to vision science. Journal of Vision, 17(2), 4. 10.1167/17.2.4 PMC5317208 28196374

[hbm26628-bib-0066] Rolls, E. T. , Deco, G. , Huang, C.‐C. , & Feng, J. (2023). Multiple cortical visual streams in humans. Cerebral Cortex, 33(7), 3319–3349. 35834308 10.1093/cercor/bhac276

[hbm26628-bib-0067] Rolls, E. T. , Deco, G. , Zhang, Y. , & Feng, J. (2023). Hierarchical organization of the human ventral visual streams revealed with magnetoencephalography. Cerebral Cortex, 33, 1–16. 10.1093/cercor/bhad318 37689834

[hbm26628-bib-0068] Saad, Z. S. , Ropella, K. M. , Cox, R. W. , & DeYoe, E. A. (2001). Analysis and use of FMRI response delays. Human Brain Mapping, 13(2), 74–93. 11346887 10.1002/hbm.1026 PMC6872085

[hbm26628-bib-0069] Saenz, M. , & Fine, I. (2010). Topographic organization of V1 projections through the corpus callosum in humans. NeuroImage, 52(4), 1224–1229. 20553894 10.1016/j.neuroimage.2010.05.060 PMC3165168

[hbm26628-bib-0070] Salimi‐Khorshidi, G. , Douaud, G. , Beckmann, C. F. , Glasser, M. F. , Griffanti, L. , & Smith, S. M. (2014). Automatic denoising of functional MRI data: Combining independent component analysis and hierarchical fusion of classifiers. NeuroImage, 90, 449–468. 24389422 10.1016/j.neuroimage.2013.11.046 PMC4019210

[hbm26628-bib-0071] Sengupta, A. , Kaule, F. R. , Guntupalli, J. S. , Hoffmann, M. B. , Häusler, C. , Stadler, J. , & Hanke, M. (2016). A studyforrest extension, retinotopic mapping and localization of higher visual areas. Scientific Data, 3(1), 160093. 27779618 10.1038/sdata.2016.93 PMC5079119

[hbm26628-bib-0072] Silson, E. H. , Steel, A. , Kidder, A. , Gilmore, A. W. , & Baker, C. I. (2019). Distinct subdivisions of human medial parietal cortex support recollection of people and places. eLife, 8, 554915. 10.7554/eLife.47391 PMC6667275 31305238

[hbm26628-bib-0073] Silson, E. H. , Steel, A. D. , & Baker, C. I. (2016). Scene‐selectivity and retinotopy in medial parietal cortex. Frontiers in Human Neuroscience, 10(August), 1–17. 27588001 10.3389/fnhum.2016.00412 PMC4988988

[hbm26628-bib-0074] Simony, E. , Honey, C. J. , Chen, J. , Lositsky, O. , Yeshurun, Y. , Wiesel, A. , & Hasson, U. (2016). Dynamic reconfiguration of the default mode network during narrative comprehension. Nature Communications, 7(1), 12141. 10.1038/ncomms12141 PMC4960303 27424918

[hbm26628-bib-0075] Smith, S. , & Nichols, T. (2009). Threshold‐free cluster enhancement: Addressing problems of smoothing, threshold dependence and localisation in cluster inference. NeuroImage, 44(1), 83–98. 18501637 10.1016/j.neuroimage.2008.03.061

[hbm26628-bib-0076] Smith, S. M. (2002). Fast robust automated brain extraction. Human Brain Mapping, 17(3), 143–155. 12391568 10.1002/hbm.10062 PMC6871816

[hbm26628-bib-0077] Smith, S. M. , Beckmann, C. F. , Andersson, J. , Auerbach, E. J. , Bijsterbosch, J. , Douaud, G. , Duff, E. , Feinberg, D. A. , Griffanti, L. , Harms, M. P. , Kelly, M. , Laumann, T. , Miller, K. L. , Moeller, S. , Petersen, S. , Power, J. , Salimi‐Khorshidi, G. , Snyder, A. Z. , Vu, A. T. , … Glasser, M. F. (2013). Resting‐state fMRI in the Human Connectome Project. NeuroImage, 80, 144–168. 23702415 10.1016/j.neuroimage.2013.05.039 PMC3720828

[hbm26628-bib-0078] Steel, A. , Billings, M. M. , Silson, E. H. , & Robertson, C. E. (2021). A network linking scene perception and spatial memory systems in posterior cerebral cortex. Nature Communications, 12(1), 2632. 10.1038/s41467-021-22848-z PMC8113503 33976141

[hbm26628-bib-0079] Steel, A. , Garcia, B. D. , Goyal, K. , Mynick, A. , & Robertson, C. E. (2023). Scene perception and visuospatial memory converge at the anterior edge of visually‐responsive cortex. The Journal of Neuroscience, 43(31), 5723–5737. 37474310 10.1523/JNEUROSCI.2043-22.2023 PMC10401646

[hbm26628-bib-0080] Steel, A. , Silson, E. H. , Garcia, B. D. , & Robertson, C. E. (2024). A retinotopic code structures the interaction between perception and memory systems. Nature Neuroscience, 27(2), 339–347. 38168931 10.1038/s41593-023-01512-3 PMC10923171

[hbm26628-bib-0081] Takemura, H. , Rokem, A. , Winawer, J. , Yeatman, J. D. , Wandell, B. A. , & Pestilli, F. (2016). A major human white matter pathway between dorsal and ventral visual cortex. Cerebral Cortex, 26(5), 2205–2214. 25828567 10.1093/cercor/bhv064 PMC4830295

[hbm26628-bib-0082] Tullo, M. G. , Almgren, H. , Van de Steen, F. , Boccia, M. , Bencivenga, F. , & Galati, G. (2023). Preferential signal pathways during the perception and imagery of familiar scenes: An effective connectivity study. Human Brain Mapping, 44(10), 3954–3971. 37219891 10.1002/hbm.26313 PMC10258540

[hbm26628-bib-0083] Van Essen, D. C. , Glasser, M. F. , Dierker, D. L. , Harwell, J. , & Coalson, T. (2012). Parcellations and hemispheric asymmetries of human cerebral cortex analyzed on surface‐based atlases. Cerebral Cortex, 22(10), 2241–2262. 22047963 10.1093/cercor/bhr291 PMC3432236

[hbm26628-bib-0084] Van Essen, D. C. , Ugurbil, K. , Auerbach, E. , Barch, D. , Behrens, T. E. J. , Bucholz, R. , Curtiss, S. W. , Oostenveld, R. , Larson‐Prior, L. J. , Schoffelen, J.‐M. , Hodge, M. R. , Cler, E. A. , Marcus, D. M. , Barch, D. M. , Yacoub, E. , Smith, S. M. , Ugurbil, K. , & Van Essen, D. C. (2012). The Human Connectome Project: A data acquisition perspective. NeuroImage, 62(4), 2222–2231. 22366334 10.1016/j.neuroimage.2012.02.018 PMC3606888

[hbm26628-bib-0085] Vincent, J. L. , Kahn, I. , Snyder, A. Z. , Raichle, M. E. , & Buckner, R. L. (2008). Evidence for a frontoparietal control system revealed by intrinsic functional connectivity. Journal of Neurophysiology, 100(6), 3328–3342. 18799601 10.1152/jn.90355.2008 PMC2604839

[hbm26628-bib-0086] Vincent, J. L. , Snyder, A. Z. , Fox, M. D. , Shannon, B. J. , Andrews, J. R. , Raichle, M. E. , & Buckner, R. L. (2006). Coherent spontaneous activity identifies a hippocampal‐parietal memory network. Journal of Neurophysiology, 96(6), 3517–3531. 16899645 10.1152/jn.00048.2006

[hbm26628-bib-0087] Virtanen, P. , Gommers, R. , Oliphant, T. E. , Haberland, M. , Reddy, T. , Cournapeau, D. , Burovski, E. , Peterson, P. , Weckesser, W. , Bright, J. , van der Walt, S. J. , Brett, M. , Wilson, J. , Millman, K. J. , Mayorov, N. , Nelson, A. R. J. , Jones, E. , Kern, R. , Larson, E. , … van Mulbregt, P. (2020). SciPy 1.0: fundamental algorithms for scientific computing in Python. Nature Methods, 17(3), 261–272. 10.1038/s41592-019-0686-2 PMC7056644 32015543

[hbm26628-bib-0088] Vossel, S. , Geng, J. J. , & Fink, G. R. (2014). Dorsal and ventral attention systems. The Neuroscientist, 20(2), 150–159. 23835449 10.1177/1073858413494269 PMC4107817

[hbm26628-bib-0089] Wang, J. , Ren, Y. , Hu, X. , Nguyen, V. T. , Guo, L. , Han, J. , & Guo, C. C. (2017). Test‐retest reliability of functional connectivity networks during naturalistic fMRI paradigms. Human Brain Mapping, 38(4), 2226–2241. 28094464 10.1002/hbm.23517 PMC6867176

[hbm26628-bib-0090] Warrington, S. , Bryant, K. L. , Khrapitchev, A. A. , Sallet, J. , Charquero‐Ballester, M. , Douaud, G. , Jbabdi, S. , Mars, R. B. , & Sotiropoulos, S. N. (2020). XTRACT – standardised protocols for automated tractography in the human and macaque brain. NeuroImage, 217, 116923. 32407993 10.1016/j.neuroimage.2020.116923 PMC7260058

[hbm26628-bib-0091] Winkler, A. M. , Ridgway, G. R. , Douaud, G. , Nichols, T. E. , & Smith, S. M. (2016). Faster permutation inference in brain imaging. NeuroImage, 141, 502–516. 27288322 10.1016/j.neuroimage.2016.05.068 PMC5035139

[hbm26628-bib-0092] Winkler, A. M. , Ridgway, G. R. , Webster, M. A. , Smith, S. M. , & Nichols, T. E. (2014). Permutation inference for the general linear model. NeuroImage, 92, 381–397. 24530839 10.1016/j.neuroimage.2014.01.060 PMC4010955

[hbm26628-bib-0093] Winkler, A. M. , Webster, M. A. , Brooks, J. C. , Tracey, I. , Smith, S. M. , & Nichols, T. E. (2016). Non‐parametric combination and related permutation tests for neuroimaging. Human Brain Mapping, 37(4), 1486–1511. 26848101 10.1002/hbm.23115 PMC4783210

[hbm26628-bib-0094] Woolrich, M. W. , Behrens, T. E. J. , Beckmann, C. F. , Jenkinson, M. , & Smith, S. M. (2004). Multilevel linear modelling for FMRI group analysis using Bayesian inference. NeuroImage, 21(4), 1732–1747. 15050594 10.1016/j.neuroimage.2003.12.023

[hbm26628-bib-0095] Woolrich, M. W. , Ripley, B. D. , Brady, M. , & Smith, S. M. (2001). Temporal autocorrelation in univariate linear modeling of FMRI data. NeuroImage, 14(6), 1370–1386. 11707093 10.1006/nimg.2001.0931

[hbm26628-bib-0096] Xiao, J. X. , Hays, J. , Ehinger, K. A. , Oliva, A. , & Torralba, A. (2010). SUN database: Large‐scale scene recognition from Abbey to Zoo. In IEEE conference on computer vision and pattern recognition (pp. 3485–3492). Los Alamitos.

[hbm26628-bib-0097] Yeo, B. T. , Krienen, F. M. , Sepulcre, J. , Sabuncu, M. R. , Lashkari, D. , Hollinshead, M. , Roffman, J. L. , Smoller, J. W. , Zöllei, L. , Polimeni, J. R. , Fischl, B. , Liu, H. , & Buckner, R. L. (2011). The organization of the human cerebral cortex estimated by intrinsic functional connectivity. Journal of Neurophysiology, 106(3), 1125–1165. 21653723 10.1152/jn.00338.2011 PMC3174820

[hbm26628-bib-0098] Zhang, Y. , Brady, M. , & Smith, S. (2001). Segmentation of brain MR images through a hidden Markov random field model and the expectation‐maximization algorithm. IEEE Transactions on Medical Imaging, 20(1), 45–57. 11293691 10.1109/42.906424

