# Supplementary material for: Mapping the functional and structural connectivity of the scene network
Source: Hum Brain Mapp. 2024 Feb 20;45(3):e26628. doi: 10.1002/hbm.26628 (PMC10878195; doi:10.1002/hbm.26628)
Supplement: Supplementary file 1 — Data S1: Supporting information. [file HBM-45-e26628-s001.docx]

# Supplementary Tables

| **Supplementary Table 1.** IDs for the 174 subjects obtained from the Human Connectome Project. Subjects marked with asterisks had missing diffusion data and were omitted from the structural connectivity analyses. Subjects marked with daggers had missing task data and were omitted from the functional localiser analyses. | | | | |
| --- | --- | --- | --- | --- |
| 100610 | 157336 | 192641 | 385046 | 770352 |
| 102311 | 158035 | 193845 | 389357 | 771354 |
| 102816 | 158136 | 195041 | 393247 | 782561 |
| 104416 | 159239 | 196144 | 395756 | 783462 |
| 105923 | 162935 | 197348 | 397760 | 789373 |
| 108323 | 164131 | 198653 | 401422 | 814649 |
| 109123 | 164636 | 199655 | 406836 | 818859 |
| 111514 | 165436 | 200210 | 412528 | 825048 |
| 114823 | 167036 | 200311 | 429040 | 826353 |
| 115017 | 167440 | 200614 | 436845 | 833249 |
| 115825 | 169040 | 201515 | 463040 | 859671 |
| 116726 | 169343 | 203418 | 467351 | 861456 |
| 118225 | 169444 | 204521 | 525541 | 871762 |
| 125525 | 169747 | 205220 | 541943 | 872764 |
| 126426 | 171633 | 209228 | 547046 | 878776 |
| 128935 | 172130 | 212419 | 550439*† | 878877 |
| 130114 | 173334 | 214019 | 562345 | 898176 |
| 130518 | 175237 | 214524 | 572045 | 899885 |
| 131217*† | 176542 | 221319 | 573249 | 901139 |
| 131722 | 177140 | 233326 | 581450 | 901442 |
| 132118 | 177645 | 239136* | 601127 | 905147 |
| 134627 | 177746 | 246133 | 617748 | 910241 |
| 134829 | 178142 | 249947 | 627549 | 926862 |
| 135124 | 178243 | 251833 | 638049 | 927359 |
| 137128 | 178647 | 257845 | 644246 | 942658 |
| 140117 | 180533 | 263436 | 654552 | 943862 |
| 144226 | 181232 | 283543 | 671855 | 958976 |
| 145834 | 182436 | 318637 | 680957 | 966975 |
| 146129 | 182739 | 320826 | 690152 | 971160 |
| 146432 | 185442 | 330324 | 706040 | 995174 |
| 146735 | 186949*† | 346137 | 724446 |  |
| 146937 | 187345 | 352738 | 725751 |  |
| 148133 | 191033 | 360030 | 732243 |  |
| 150423* | 191336 | 365343 | 751550 |  |
| 155938 | 191841 | 380036 | 757764 |  |
| 156334 | 192439 | 381038 | 765864 |  |

| **Supplementary Table 2.** MNI coordinates and size of functionally defined scene-selective regions of interest in each dataset. | | | | | | | | |  |
| --- | --- | --- | --- | --- | --- | --- | --- | --- | --- |
| Dataset | Region | Hemi-sphere | Target Size | Actual Size | Threshold (Z-score) | Peak Grayordinate (mm) | | | |
|  |  |  |  |  |  | x | y | z | |
| Game of Thrones | PPA | Left | 2000 mm^3^ | 2000 mm^3^ | 8.12 | -24 | -50 | -8 | |
|  |  | Right |  | 2000 mm^3^ | 8.85 | 28 | -42 | -8 | |
|  | RSC | Left |  | 2000 mm^3^ | 4.89 | -18 | -52 | 6 | |
|  |  | Right |  | 2000 mm^3^ | 5.92 | 18 | -50 | 8 | |
|  | OPA | Left |  | 2000 mm^3^ | 7.55 | -32 | -90 | 16 | |
|  |  | Right |  | 2000 mm^3^ | 7.87 | 34 | -84 | 20 | |
| Study Forrest | PPA | Left | 2000 mm^3^ | 2000 mm^3^ | 4.88 | -26 | -50 | -10 | |
|  |  | Right |  | 2000 mm^3^ | 5.07 | 24 | -46 | -10 | |
|  | RSC | Left |  | 2000 mm^3^ | 4.32 | -12 | -54 | 8 | |
|  |  | Right |  | 2000 mm^3^ | 4.39 | 10 | -50 | 6 | |
|  | OPA | Left |  | 1992 mm^3^ | 4.28 | -26 | -88 | 30 | |
|  |  | Right |  | 2000 mm^3^ | 4.44 | 34 | -78 | 18 | |
| HCP | PPA | Left | 500 mm^2^ | 494 mm^2^ | 15.13 | -30 | -49 | -8 | |
|  |  | Right |  | 500 mm^2^ | 15.40 | 32 | -46 | -8 | |
|  | RSC | Left |  | 500 mm^2^ | 10.09 | -18 | -60 | 13 | |
|  |  | Right |  | 501 mm^2^ | 11.80 | 19 | -55 | 11 | |
|  | OPA | Left |  | 500 mm^2^ | 15.51 | -30 | -88 | 15 | |
|  |  | Right |  | 506 mm^2^ | 15.40 | 35 | -80 | 20 | |

| **Supplementary Table 3.** MNI y-axis coordinate of split between posterior and anterior subdivisions of PPA region, and size of subdivisions. | | | | |
| --- | --- | --- | --- | --- |
| Dataset | Hemisphere | y-axis coordinate of split (mm) | pPPA size | aPPA size |
| Game of Thrones | Left | -48 | 1096 mm^3^ | 904 mm^3^ |
|  | Right | -44 | 864 mm^3^ | 1136 mm^3^ |
| StudyForrest | Left | -46 | 920 mm^3^ | 1080 mm^3^ |
|  | Right | -46 | 968 mm^3^ | 1032 mm^3^ |
| HCP | Left | -51 | 248 mm^2^ | 246 mm^2^ |
|  | Right | -48 | 250 mm^2^ | 250 mm^2^ |

# Supplementary Figures


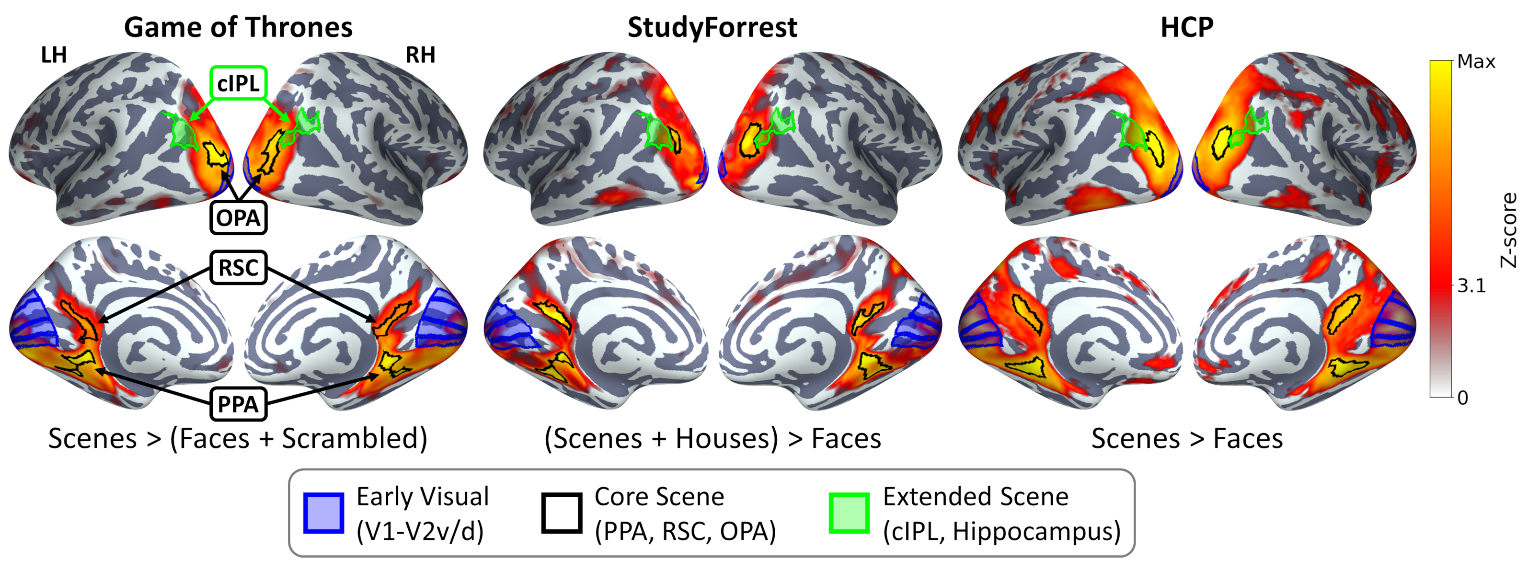


**Supplementary Figure 1.** Locations of regions of interest in each dataset. Core scene regions (Parahippocampal Place Area [PPA], Retrosplenial Complex [RSC], Occipital Place Area [OPA]) were defined from the functional contrasts annotated below each panel. Statistical overlays show Z-score maps for the specified contrast, displayed unthresholded but appearing semi-transparent below Z = 3.1 (one-tailed *p* = .001). Annotations also indicate locations of early visual regions (V1v, V1d, V2v, V2d) and extended scene regions comprising the caudal Inferior Parietal Lobule (cIPL) and hippocampus (not pictured).


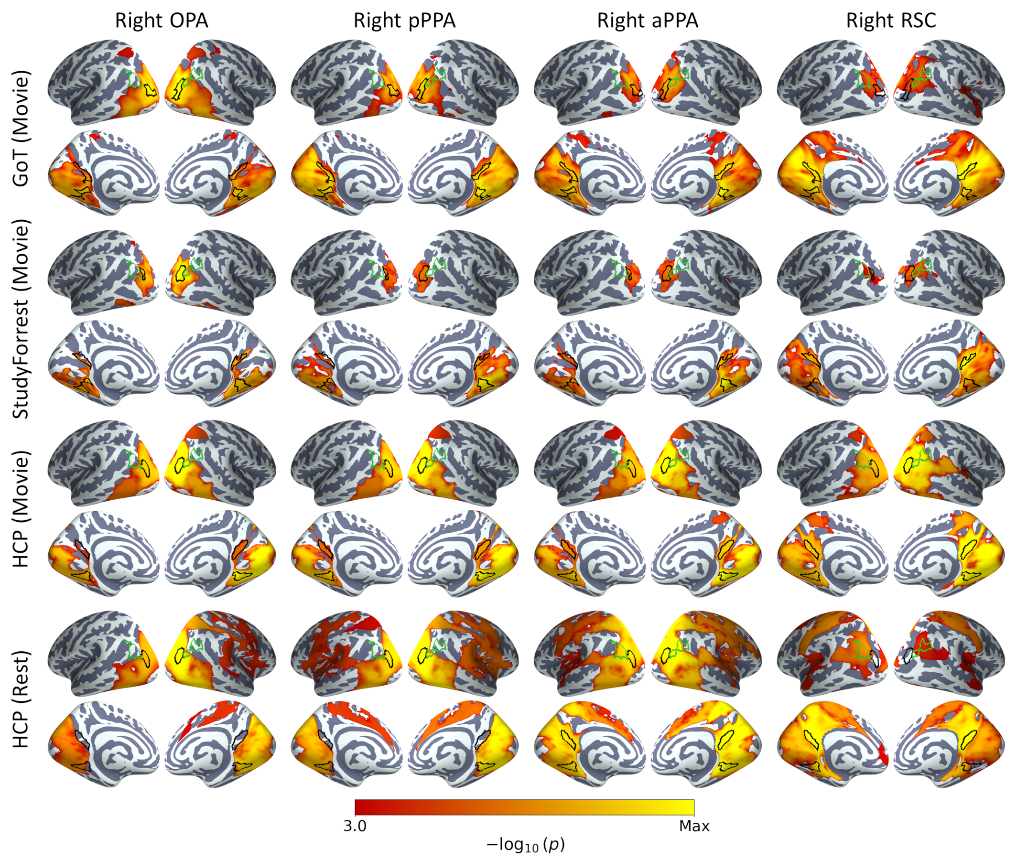


**Supplementary Figure 2.** Seed-based functional connectivity between right hemisphere core scene regions and cortical grey matter. Statistical overlays illustrate FWER-corrected TFCE *p*-values for one-sample tests of functional connectivity correlations against zero over subjects. Annotations indicate locations of core scene (OPA, PPA, RSC; black outlines) and cIPL (green outlines) regions.


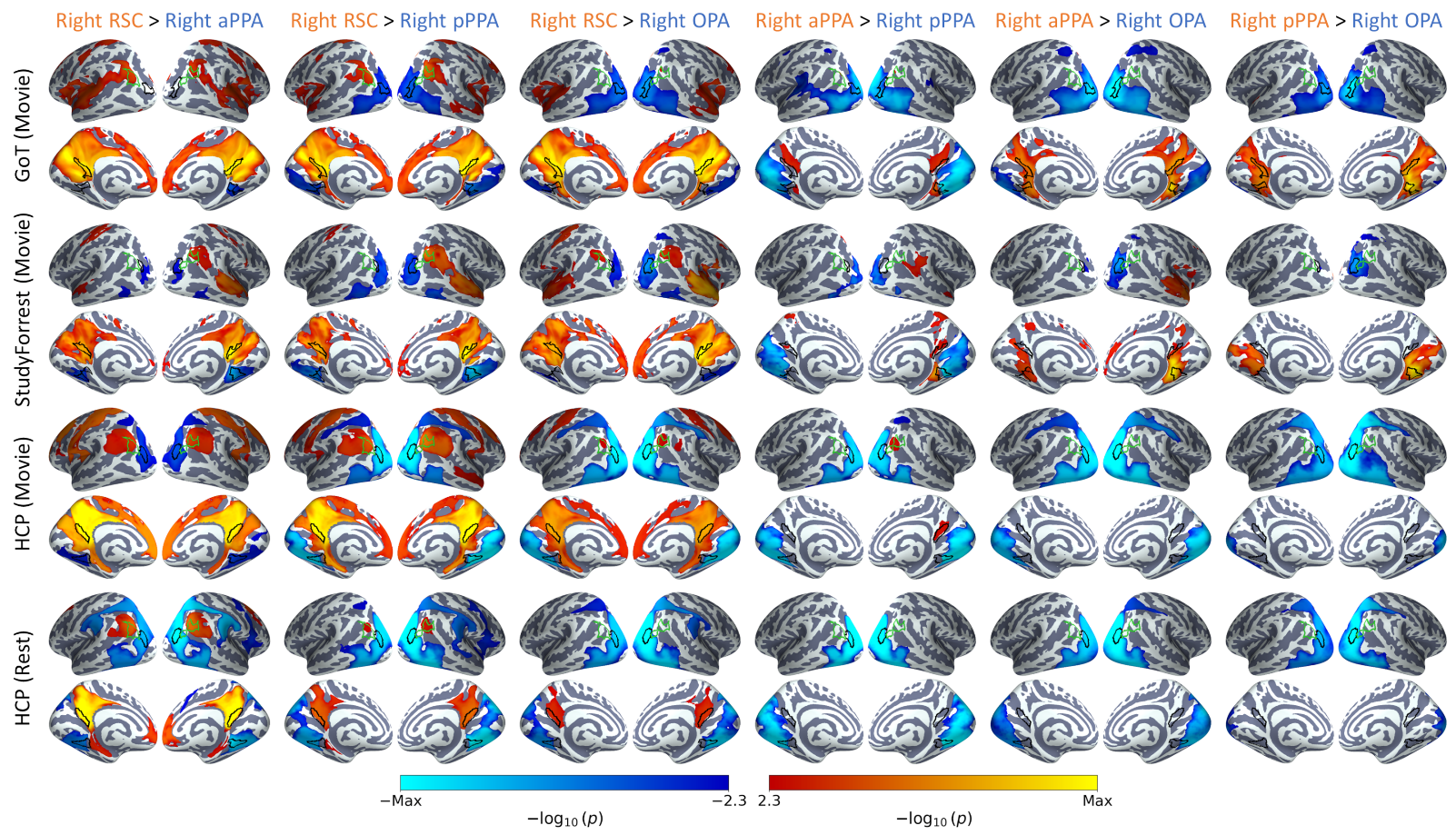


**Supplementary Figure 3.** Contrasts of seed-based functional connectivity measured between right hemisphere core scene regions and cortical grey matter. Statistical overlays illustrate FWER-corrected TFCE *p*-values for paired-sample tests of functional connectivity correlations between seed regions over subjects. Annotations indicate locations of core scene (OPA, PPA, RSC; black outlines) and cIPL (green outlines) regions.


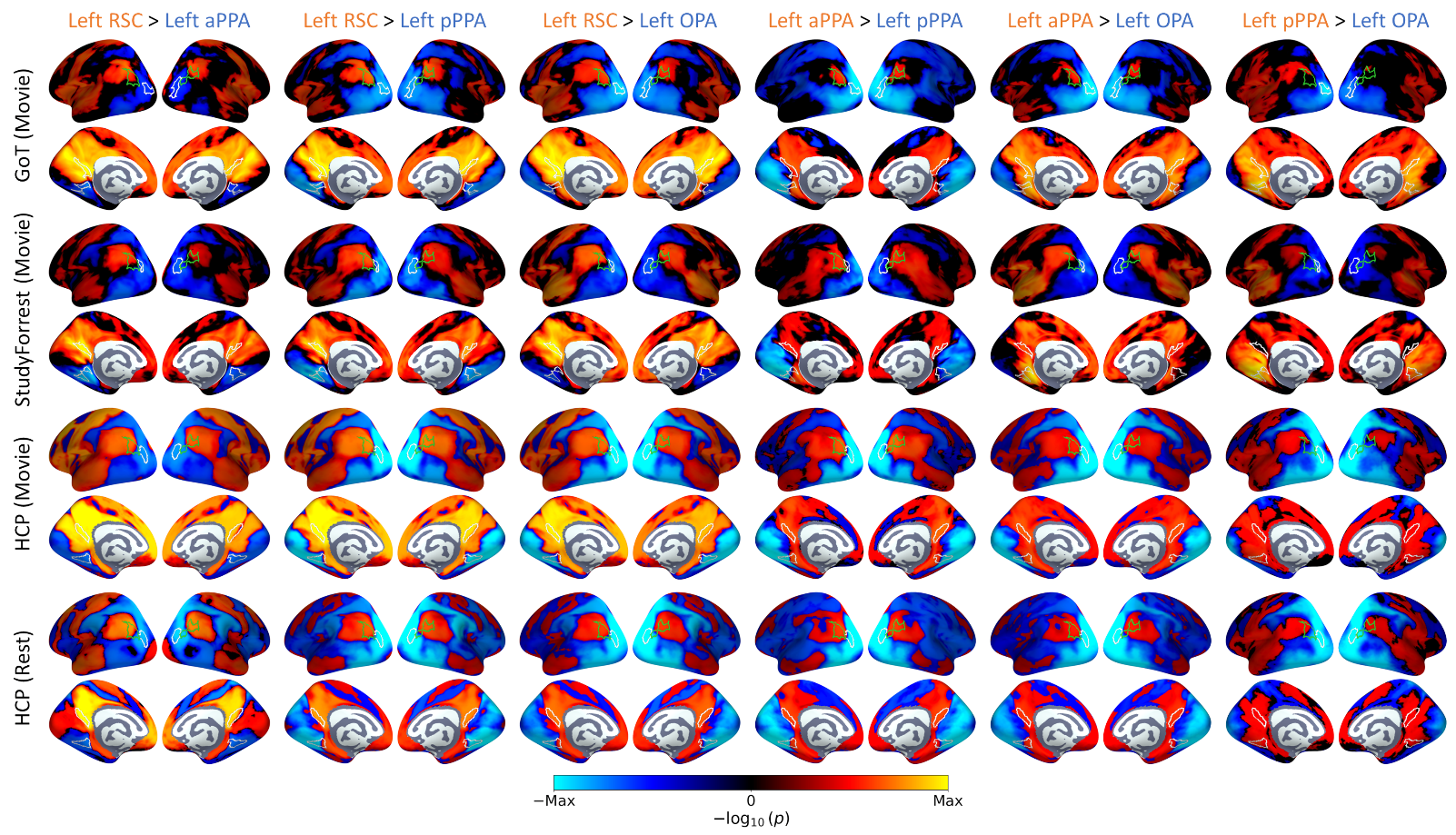


**Supplementary Figure 4.** Unthresholded contrasts of seed-based functional connectivity measured between left hemisphere core scene regions and cortical grey matter. Statistical overlays illustrate FWER-corrected TFCE *p*-values for paired-sample tests of functional connectivity correlations between seed regions over subjects. Annotations indicate locations of core scene (OPA, PPA, RSC; white outlines) and cIPL (green outlines) regions.


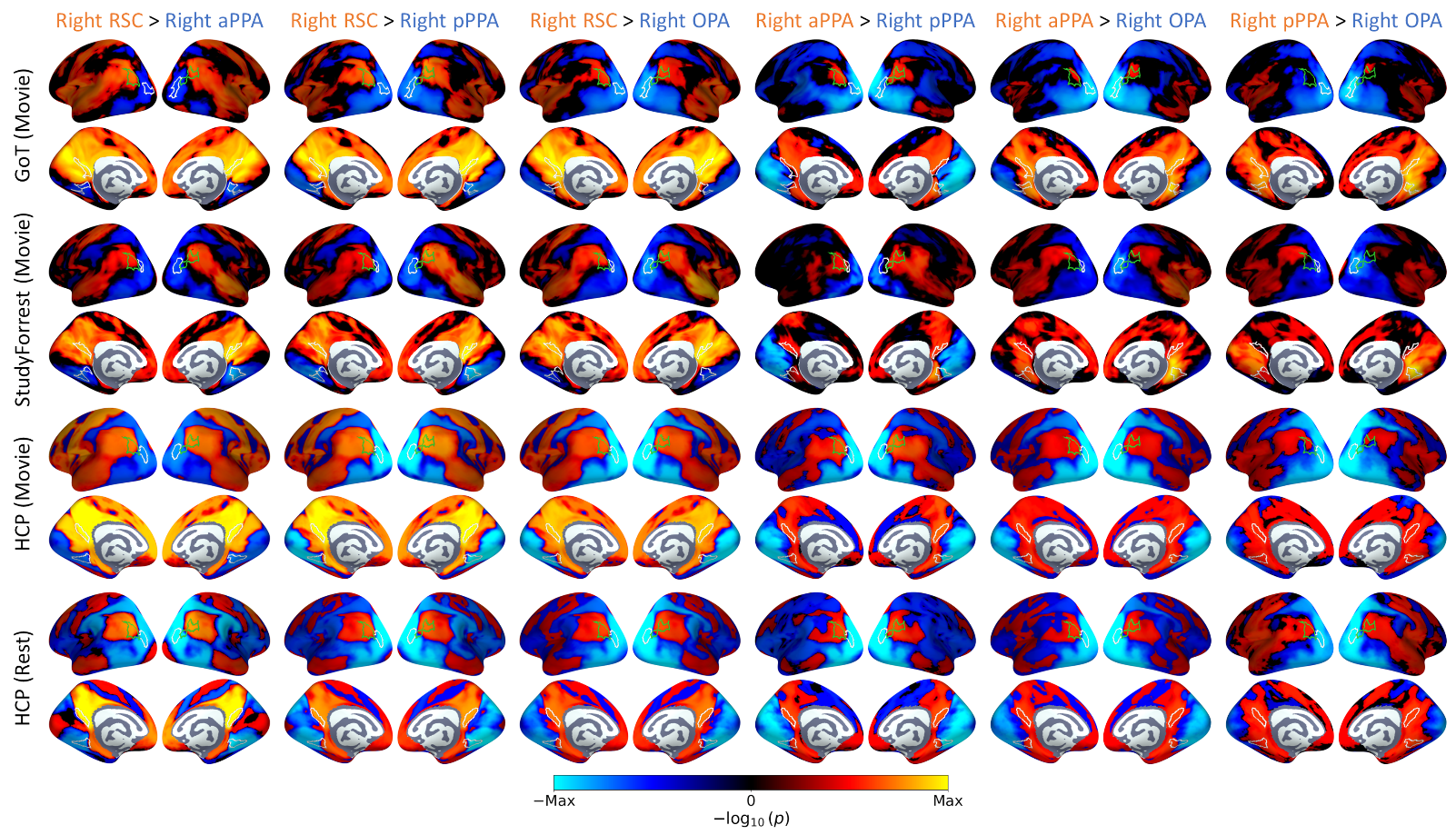


**Supplementary Figure 5.** Unthresholded contrasts of seed-based functional connectivity measured between right hemisphere core scene regions and cortical grey matter. Statistical overlays illustrate FWER-corrected TFCE *p*-values for paired-sample tests of functional connectivity correlations between seed regions over subjects. Annotations indicate locations of core scene (OPA, PPA, RSC; white outlines) and cIPL (green outlines) regions.


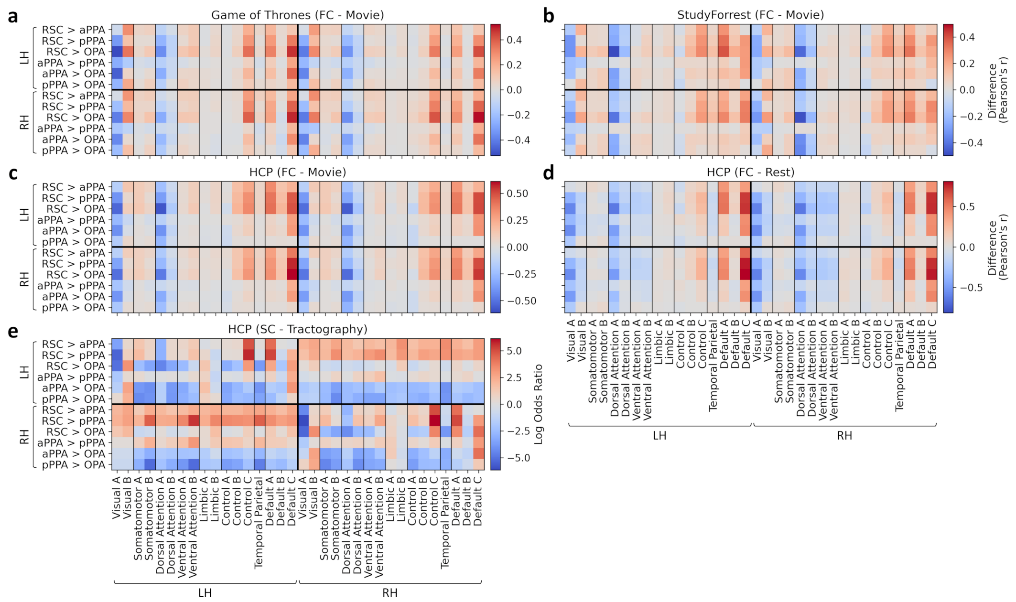


**Supplementary Figure 6.** Contrasts of connectivity measured between core scene regions and 17 resting-state networks (cf. Figure 4). Matrices illustrate group average values. Contrasts of functional connectivity are measured by the difference in correlation values between a given pair or regions. Contrasts of structural connectivity are measured by converting the connection probabilities to odds then taking the log odds ratio between a given pair of regions.


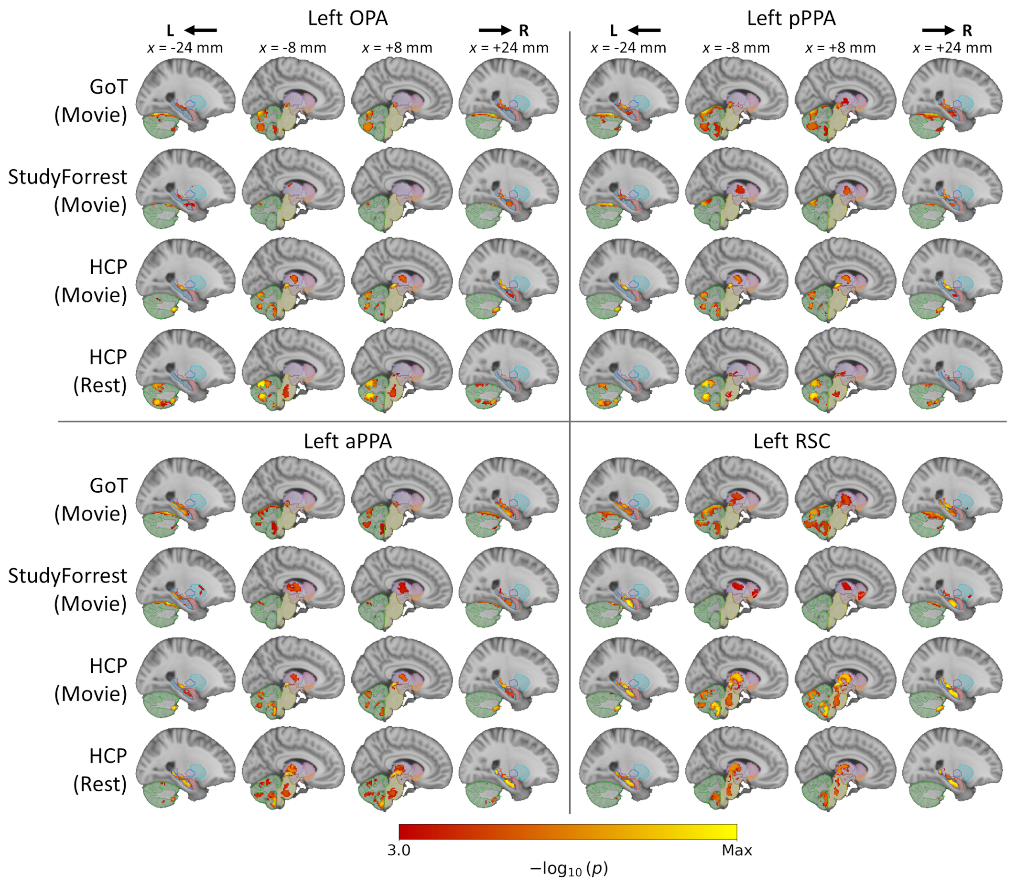


**Supplementary Figure 7.** Seed-based functional connectivity between left hemisphere core scene regions and subcortical grey matter. Statistical overlays illustrate FWER-corrected TFCE *p*-values for one-sample tests of functional connectivity correlations against zero over subjects.


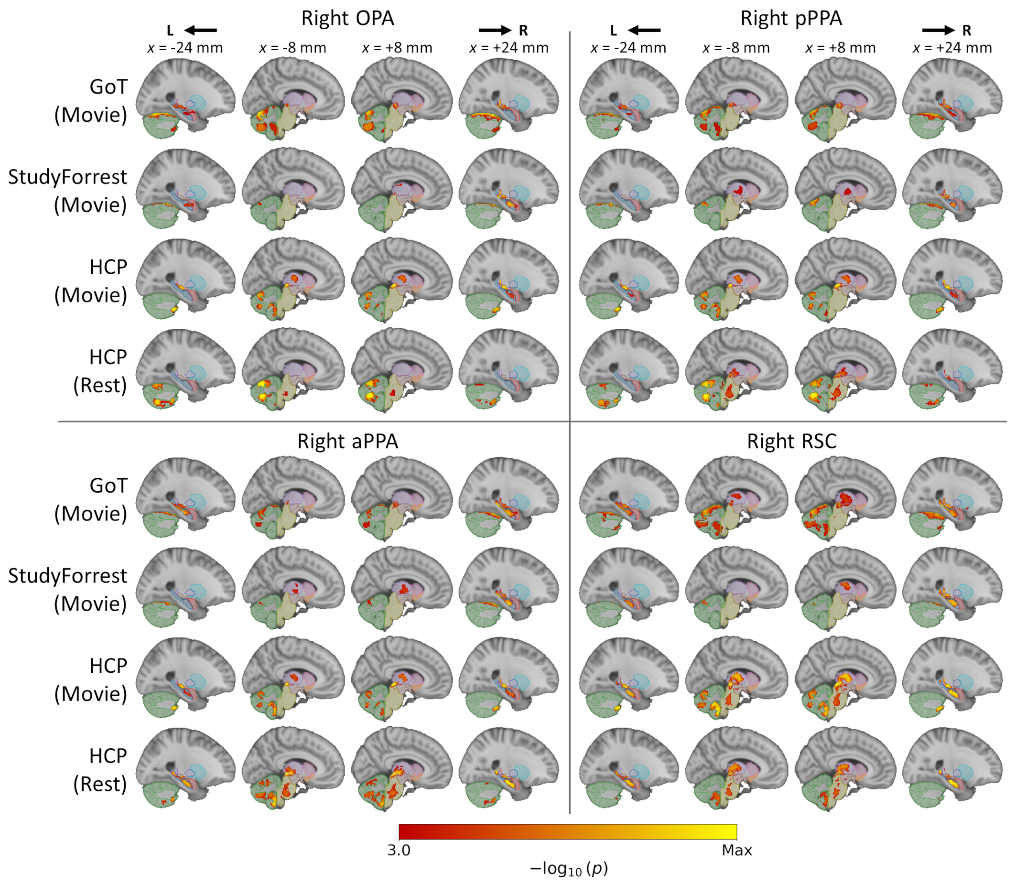


**Supplementary Figure 8.** Seed-based functional connectivity between right hemisphere core scene regions and subcortical grey matter. Statistical overlays illustrate FWER-corrected TFCE *p*-values for one-sample tests of functional connectivity correlations against zero over subjects.

**
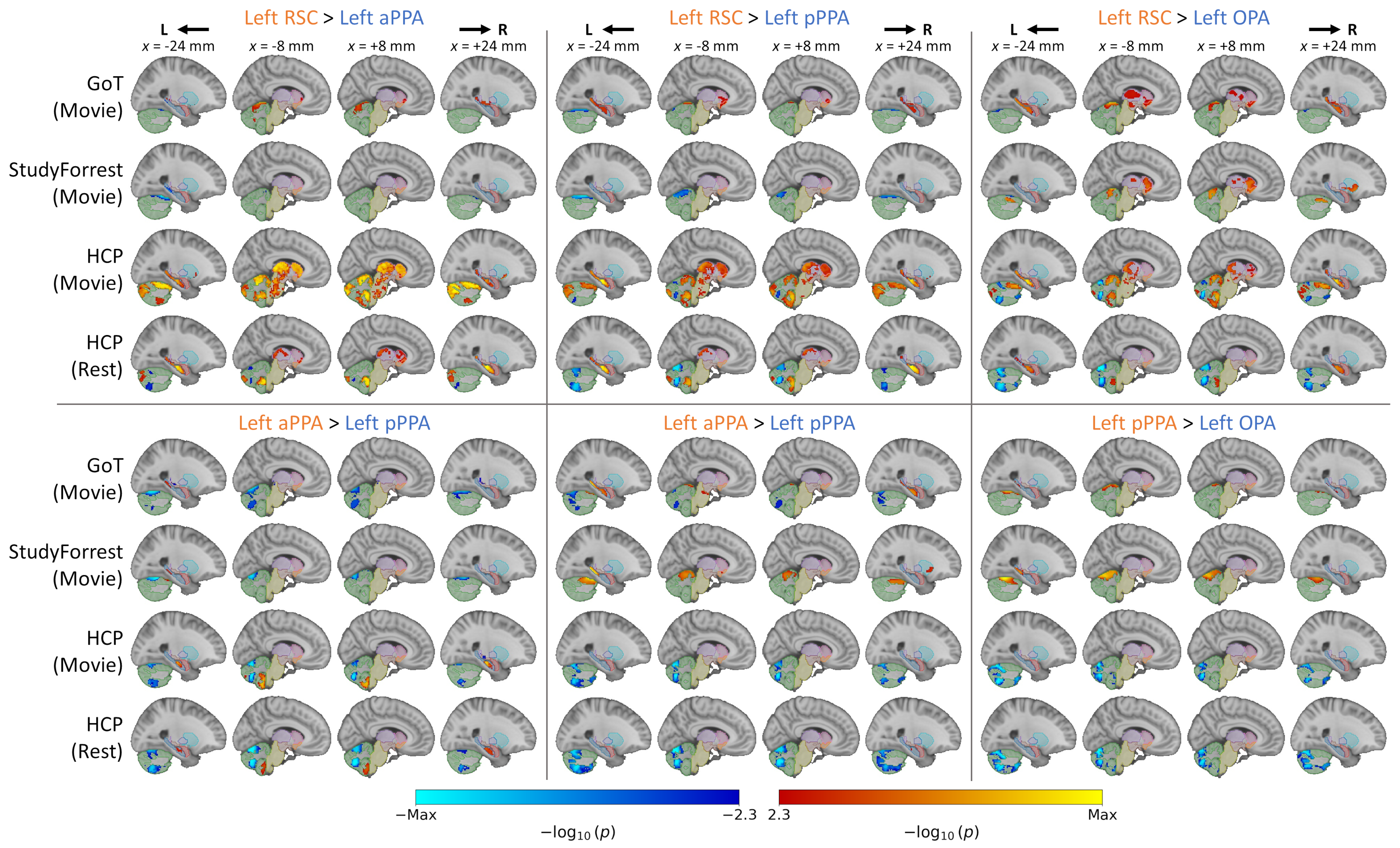
**

**Supplementary Figure 9.** Contrasts of seed-based functional connectivity measured between left hemisphere core scene regions and subcortical grey matter. Statistical overlays illustrate FWER-corrected TFCE *p*-values for paired-sample tests of functional connectivity correlations between seed regions over subjects.

**
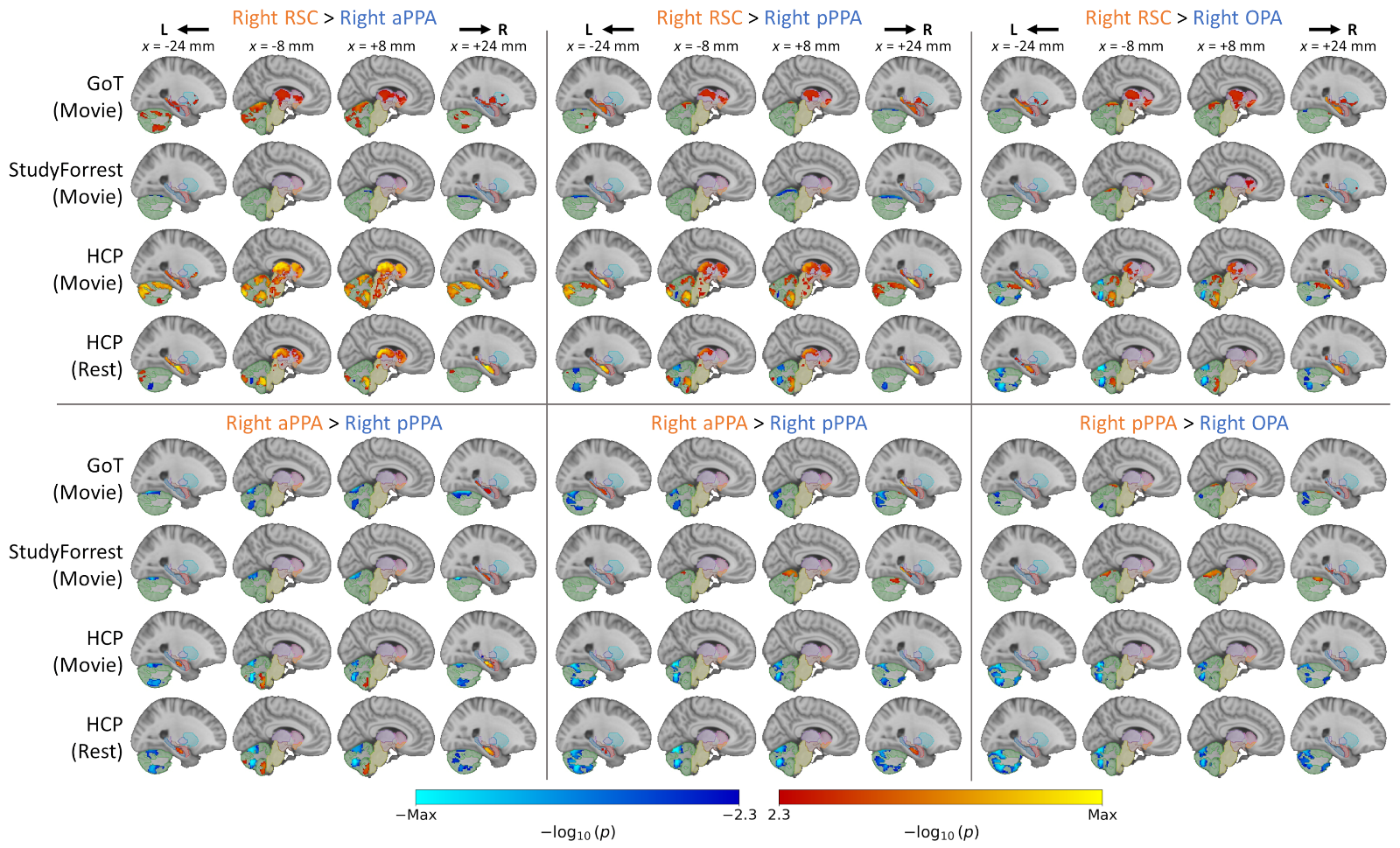
**

**Supplementary Figure 10.** Contrasts of seed-based functional connectivity measured between right hemisphere core scene regions and subcortical grey matter. Statistical overlays illustrate FWER-corrected TFCE *p*-values for paired-sample tests of functional connectivity correlations between seed regions over subjects.


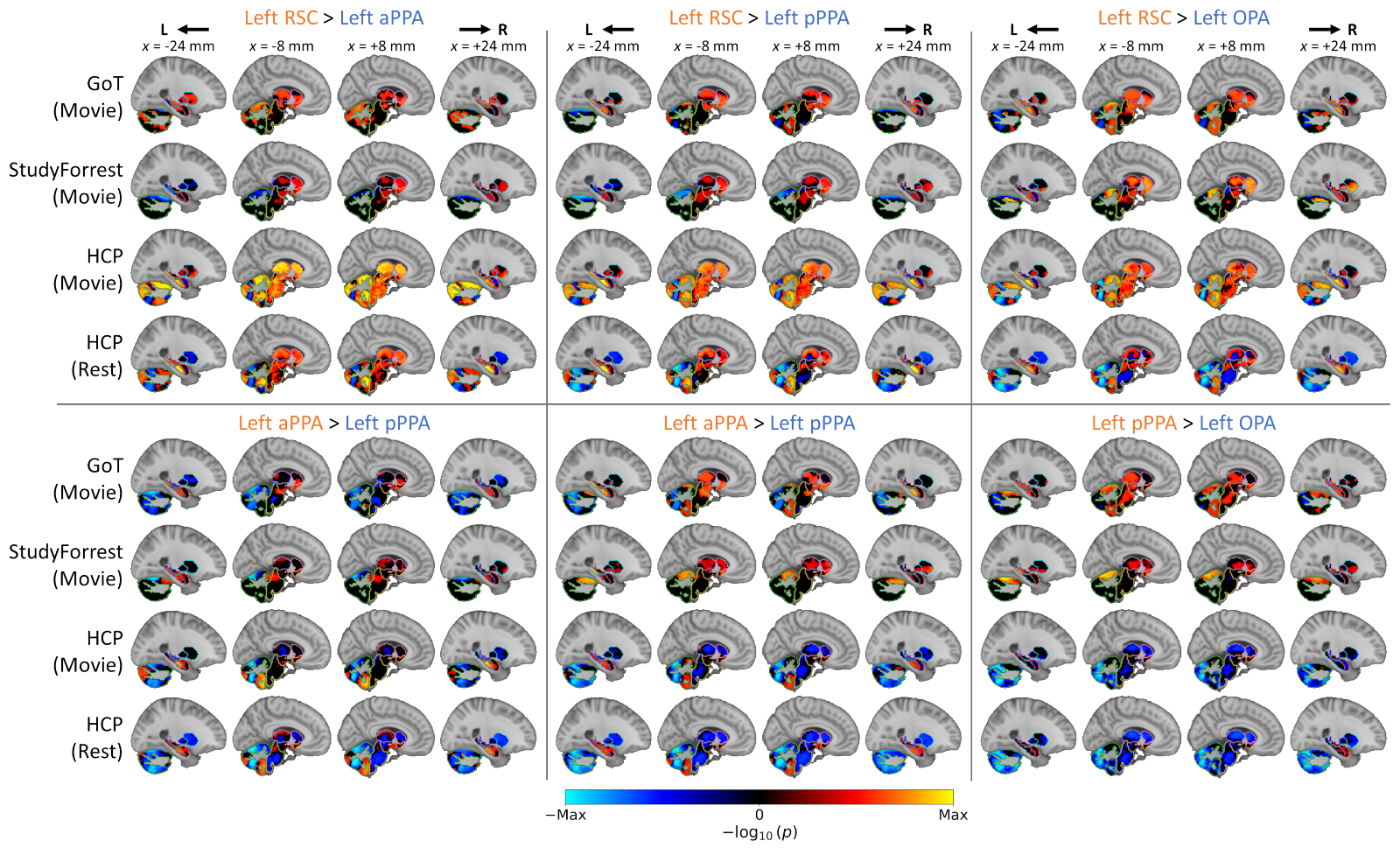


**Supplementary Figure 11.** Unthresholded contrasts of seed-based functional connectivity measured between left hemisphere core scene regions and subcortical grey matter. Statistical overlays illustrate FWER-corrected TFCE *p*-values for paired-sample tests of functional connectivity correlations between seed regions over subjects.


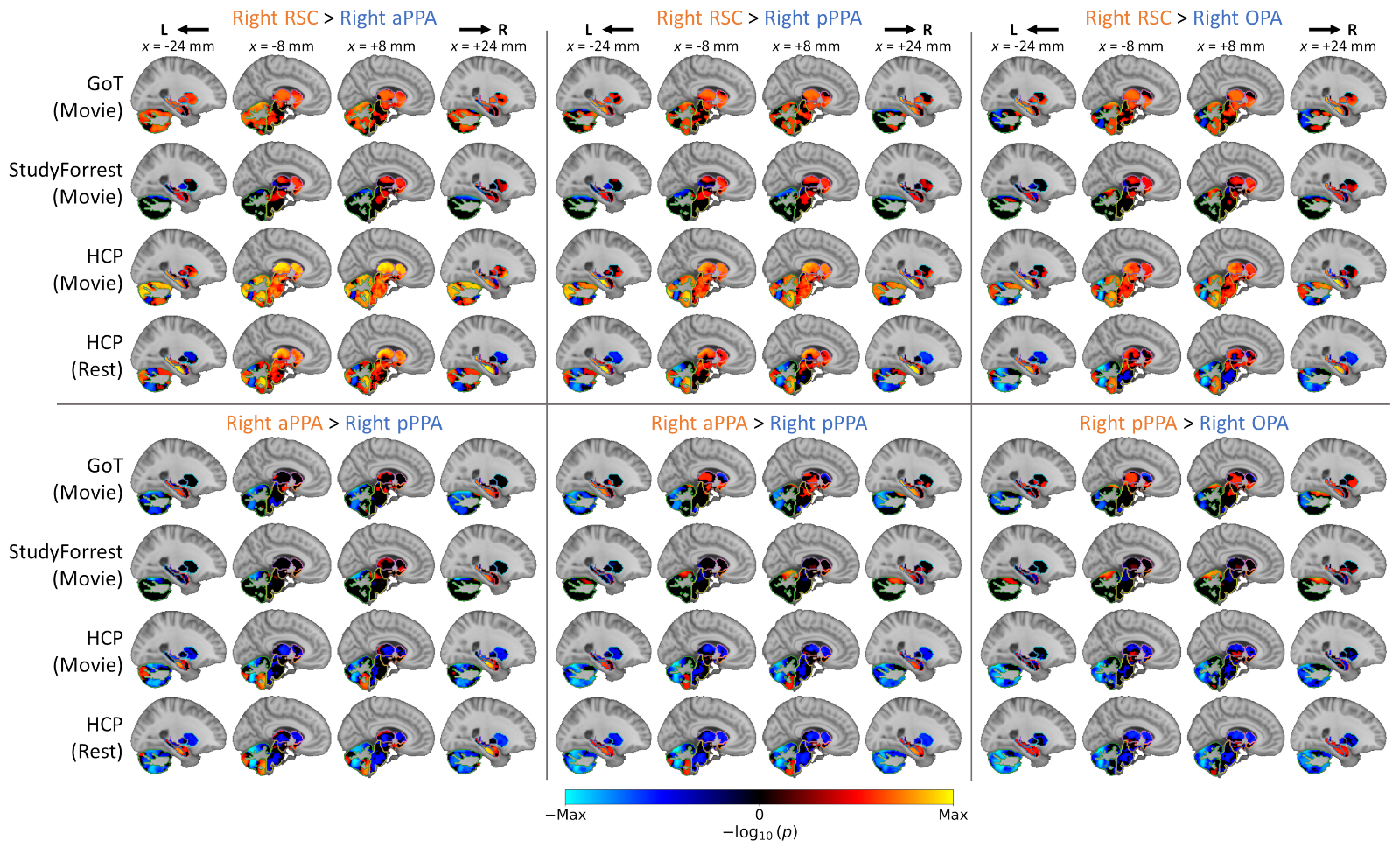


**Supplementary Figure 12.** Unthresholded contrasts of seed-based functional connectivity measured between right hemisphere core scene regions and subcortical grey matter (cf. Supplementary Figure 9). Statistical overlays illustrate FWER-corrected TFCE *p*-values for paired-sample tests of functional connectivity correlations between seed regions over subjects.


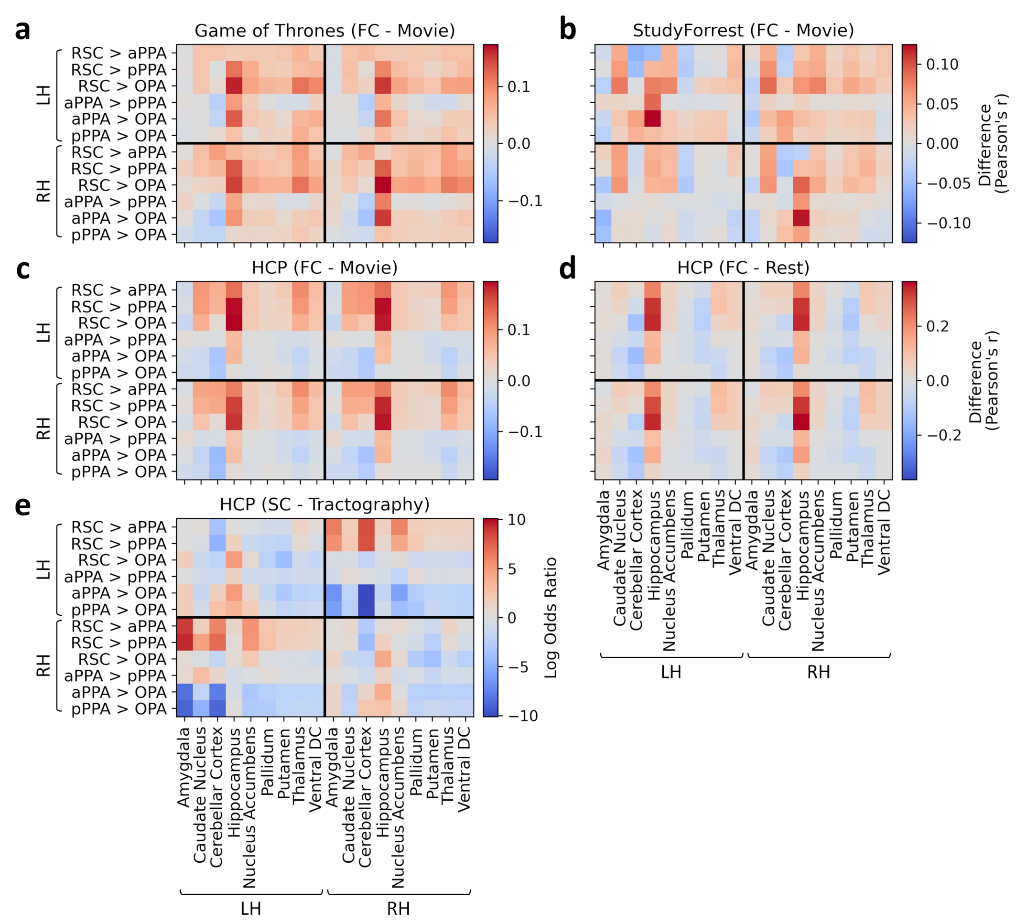


**Supplementary Figure 13.** Contrasts of connectivity measured between core scene regions and subcortical structures (cf. Figure 5). Matrices illustrate group average values. Contrasts of functional connectivity are measured by the difference in correlation values between a given pair or regions. Contrasts of structural connectivity are measured by converting the connection probabilities to odds then taking the log odds ratio between a given pair of regions.


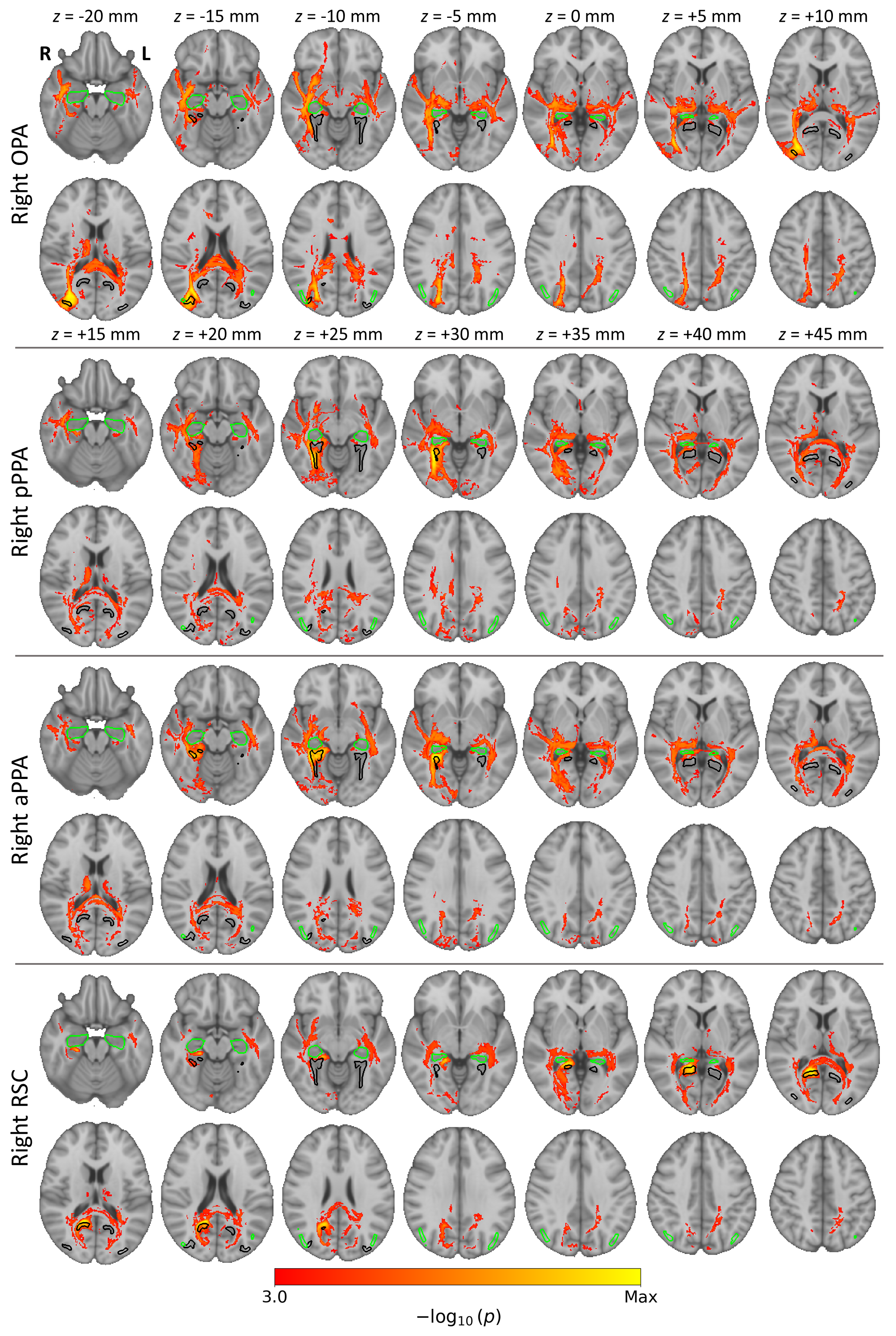


**Supplementary Figure 14.** Seed-based tractography from right hemisphere core scene regions. Statistical overlays illustrate FWER-corrected TFCE *p*-values for one-sample tests of connection probabilities against zero over subjects. Annotations indicate locations of core (OPA, PPA, RSC; black outlines) and extended (cIPL, hippocampus; green outlines) scene regions.


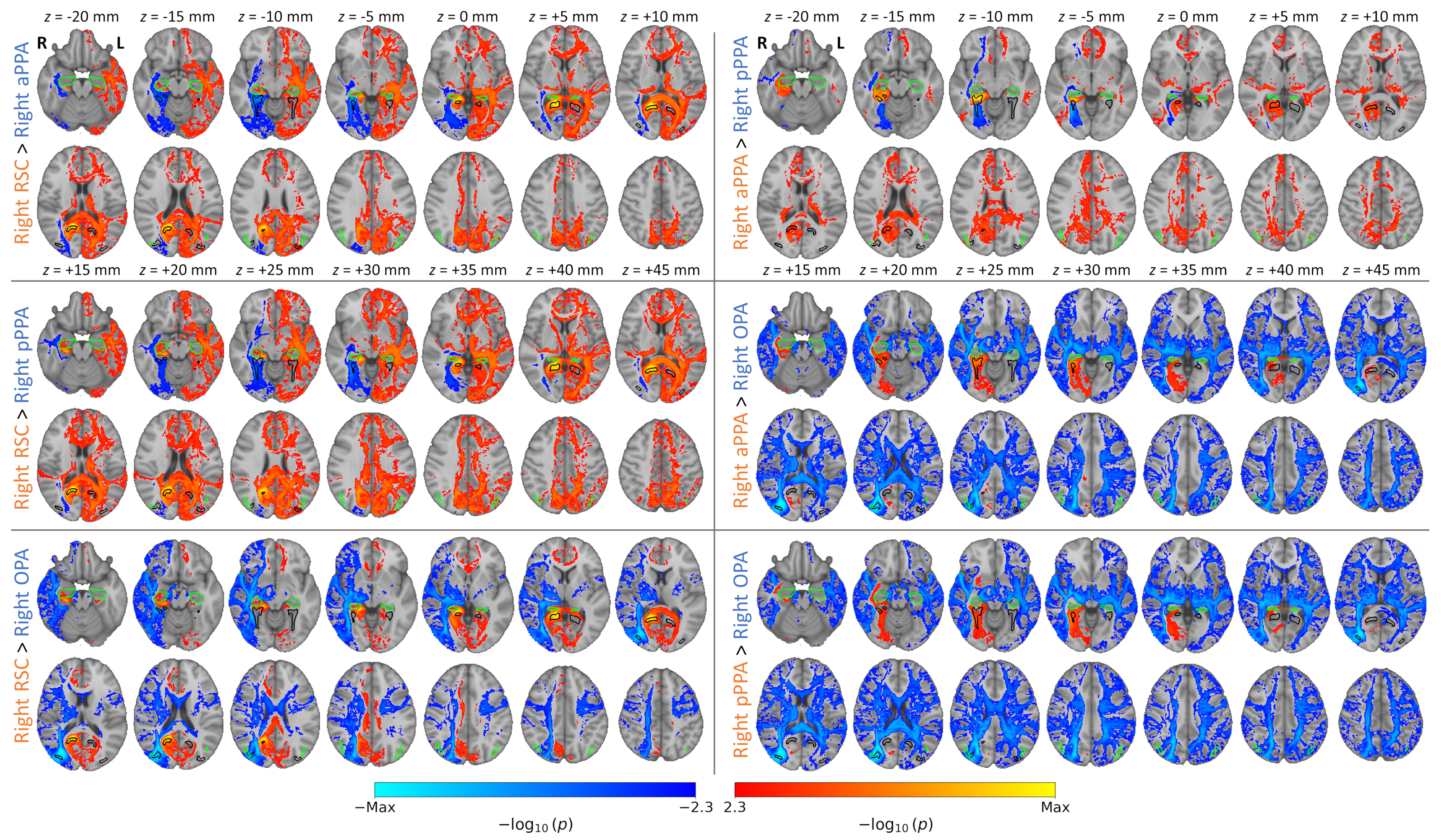


**Supplementary Figure 15.** Contrasts of seed-based tractography between right hemisphere core scene regions. Statistical overlays illustrate FWER-corrected TFCE *p*-values for paired-sample tests of connection probabilities between seed regions over subjects. Annotations indicate locations of core (OPA, PPA, RSC; black outlines) and extended (cIPL, hippocampus; green outlines) scene regions.


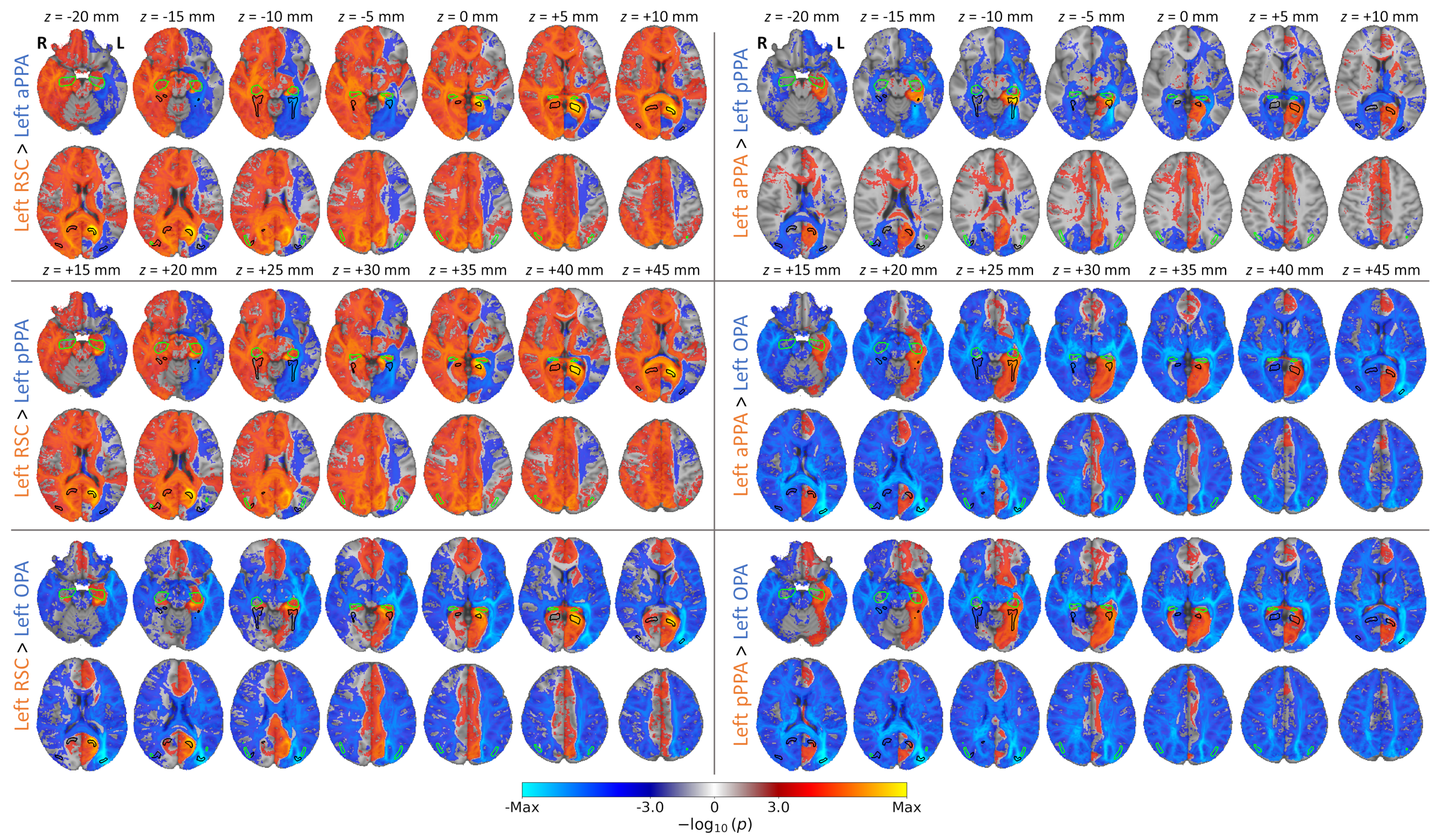


**Supplementary Figure 16.** Unthresholded contrasts of seed-based tractography between left hemisphere core scene regions. Statistical overlays illustrate FWER-corrected TFCE *p*-values for paired-sample tests of connection probabilities between seed regions over subjects. Overlays are displayed unthresholded but appear semi-transparent below -log_10_(*p*) = 3 (*p* = .001). Annotations indicate locations of core (OPA, PPA, RSC; black outlines) and extended (cIPL, hippocampus; green outlines) scene regions.


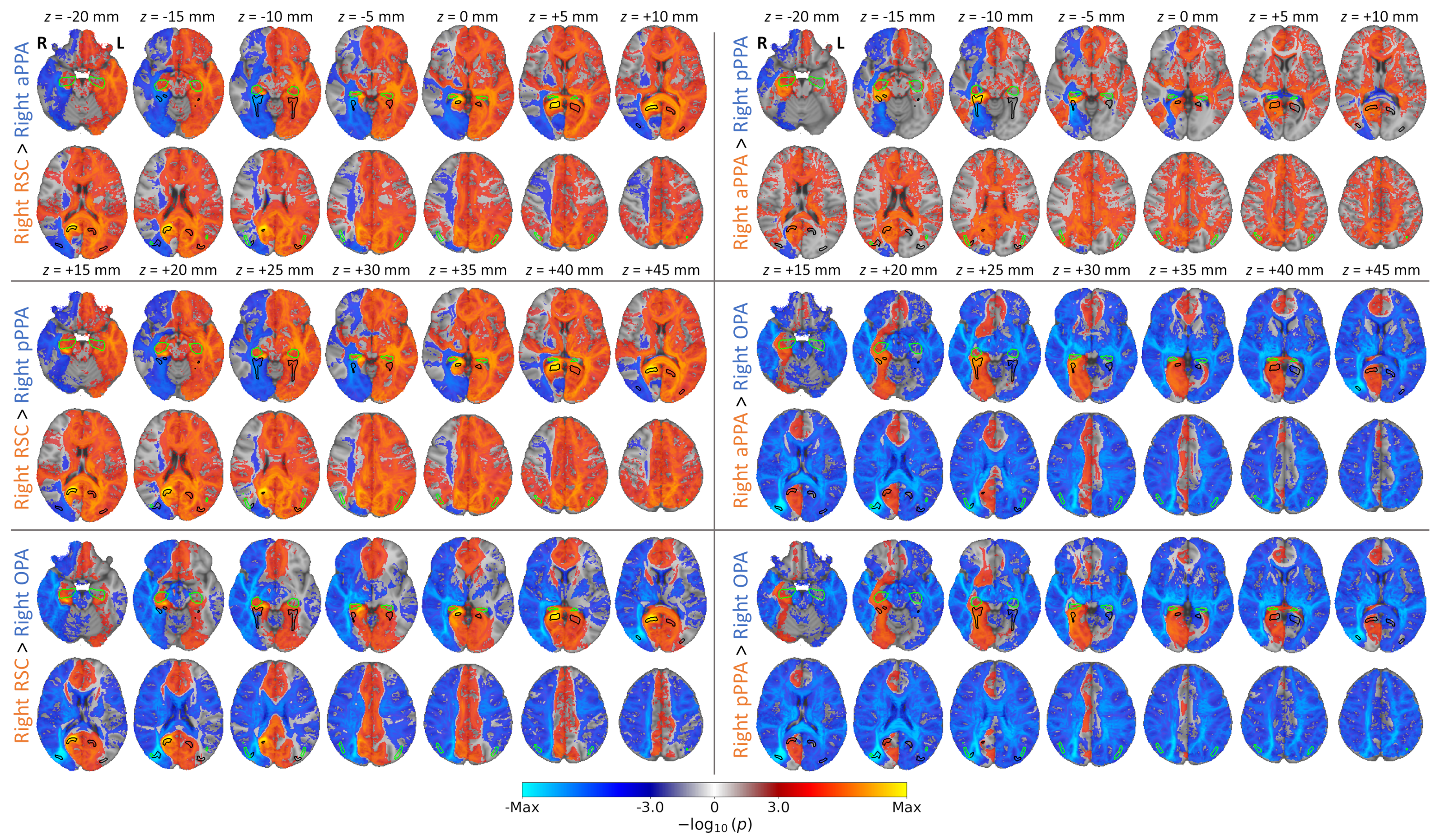


**Supplementary Figure 17.** Unthresholded contrasts of seed-based tractography between right hemisphere core scene regions. Statistical overlays illustrate FWER-corrected TFCE *p*-values for paired-sample tests of connection probabilities between seed regions over subjects. Overlays are displayed unthresholded but appear semi-transparent below -log_10_(*p*) = 3 (*p* = .001). Annotations indicate locations of core (OPA, PPA, RSC; black outlines) and extended (cIPL, hippocampus; green outlines) scene regions.

**
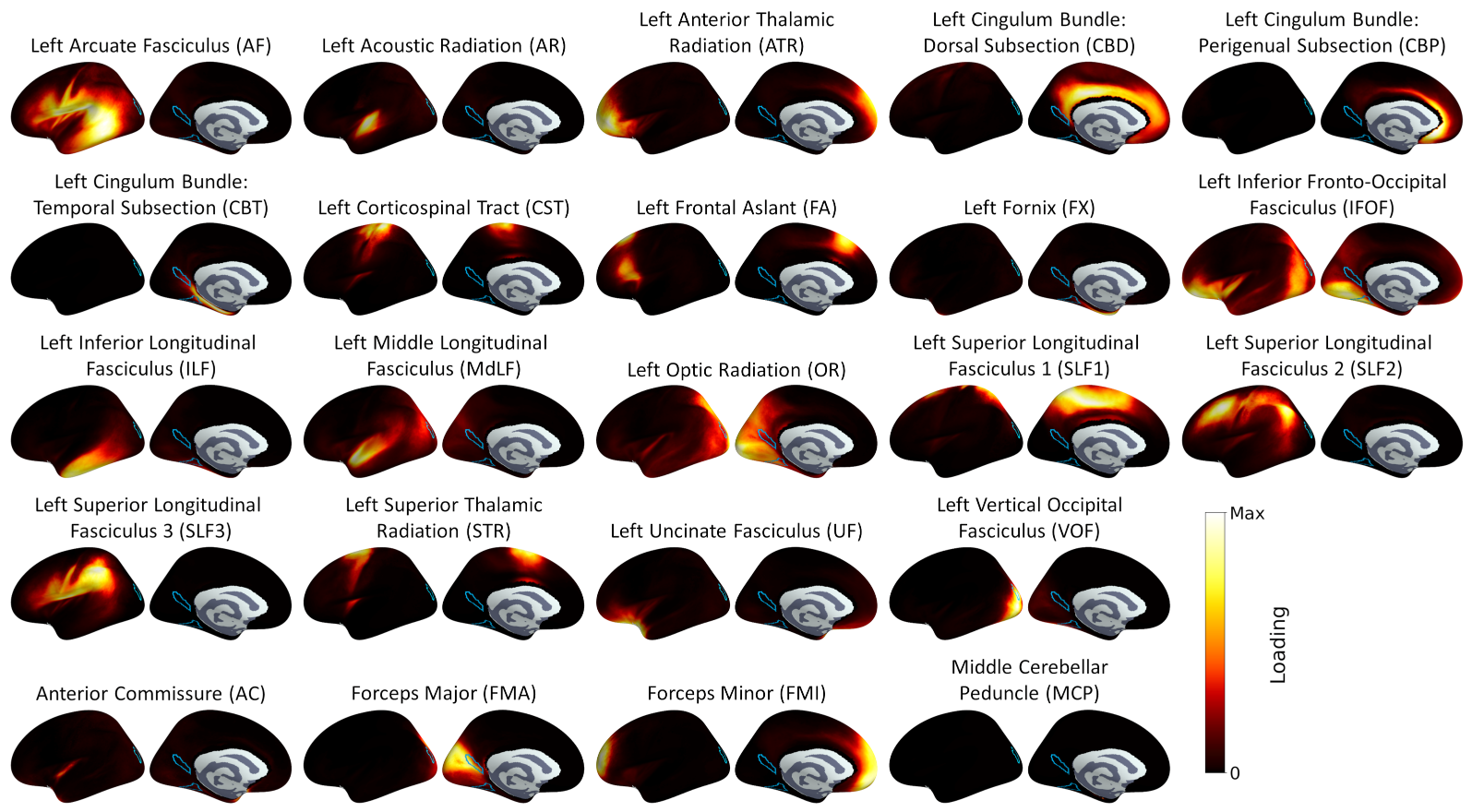
**

**Supplementary Figure 18.** Whole-brain tractography blueprints for the white matter tracts reconstructed with the XTRACT toolbox. Higher loadings indicate a greater probability of a given tract terminating at that location along the grey/white matter boundary. Plots show blueprints for all left hemisphere and interhemispheric tracts (right hemisphere tracts are not pictured). Blue outlines indicate the locations of the core scene regions (PPA, RSC, OPA) defined from the HCP dataset.

# Supplementary Videos

**Supplementary Video 1.** 3D renderings of seed-based tractography from core scene regions. Statistical overlays illustrate FWER-corrected TFCE *p*-values for one-sample tests of connection probabilities against zero over subjects.

**Supplementary Video 2.** 3D renderings of contrasts of seed-based tractography between left hemisphere core scene regions. Statistical overlays illustrate FWER-corrected TFCE *p*-values for paired-sample tests of connection probabilities between seed regions over subjects.

**Supplementary Video 3.** 3D renderings of contrasts of seed-based tractography between right hemisphere core scene regions. Statistical overlays illustrate FWER-corrected TFCE *p*-values for paired-sample tests of connection probabilities between seed regions over subjects.
